# New Caffeoylquinic Acid Derivatives and Flavanone Glycoside from the Flowers of *Chrysanthemum morifolium* and Their Bioactivities

**DOI:** 10.3390/molecules24050850

**Published:** 2019-02-28

**Authors:** Peng-Fei Yang, Ya-Nan Yang, Chun-Yu He, Zhi-Fei Chen, Qi-Shan Yuan, Sheng-Chen Zhao, Yu-Feng Fu, Pei-Cheng Zhang, Duo-Bin Mao

**Affiliations:** 1 College of Food and Biological Engineering, Zhengzhou University of Light Industry, Zhengzhou 450000, China; pf_yang@zzuli.edu.cn (P.-F.Y.); heworks@163.com (C.-Y.H.); 2 State Key Laboratory of Bioactive Substance and Function of Natural Medicines, Institute of Materia Medica, Chinese Academy of Medical Sciences and Peking Union Medical College, Beijing 100050, China; yyn@imm.ac.cn; 3 Technology Center, China Tobacco Henan Industrial Co. Ltd., Zhengzhou 450000, China; zhifei1111@126.com (Z.-F.C.); xyyqs@126.com (Q.-S.Y.); 13623810925@126.com (S.-C.Z.); fuyufeng89@163.com (Y.-F.F.)

**Keywords:** *Chrysanthemum morifolium*, caffeoylquinic acid, flavanone glycoside, neuroprotective activity

## Abstract

The *Chrysanthemum morifolium* flower is widely used in China and Japan as a food, beverage, and medicine for many diseases. In our work, two new caffeoylquinic acid derivatives (**1, 2**), a new flavanone glycoside (**3**), and six reported flavanones (**4**–**9**) were isolated and identified from the flowers of *C. morifolium*. The chemical structures of all isolates were elucidated by the analysis of comprehensive spectroscopic data as well as by comparison with previously reported data. The isolated constituents **1**–**8** were evaluated for their neuroprotective activity, and compounds **3** and **4** displayed neuroprotective effects against hydrogen peroxide-induced neurotoxicity in human neuroblastoma SH-SY5Y cells.

## 1. Introduction

**Figure 1:**
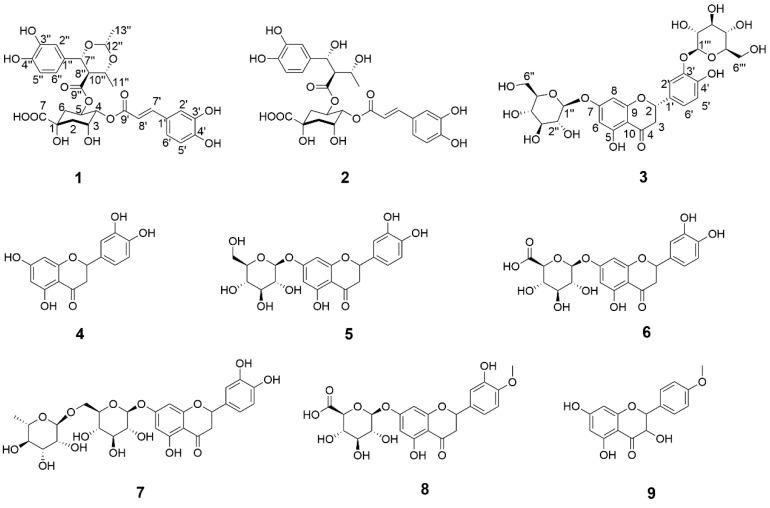
Chemical structures of compounds **1**–**9**.

The flowers of *Chrysanthemum morifolium* Ramat., *Flos Chrysanthemi,* which are cultivated in Zhejiang Province as ‘Hangbaiju′ (HJ), have been widely and traditionally consumed in China as a medicinal and edible cognate for about 2000 years. As a well-known herb medicine, HJ is used for its efficacy in dispersing cold, eliminating heat, and improving liver function [1]. Simultaneously, tea made from HJ with hot or boiling water is a popular beverage that cools fever and heightens eyesight [2]. The biological activities of HJ have been exhaustively reported and include cardiovascular protection [3], anti-oxidation [4,5,6], anti-human immune deficiency viruses activity [7,8], anti-inflammatory activity [9], vasorelaxant activity [10], neuroprotective activity [11], anti-cancer activity [12,13], hepatoprotective effects [14,15], aldose reductase inhibition [16,17], and anti-mutagenesis activity [18]. Because of its diversiform and appealing pharmacological activities, HJ has been the subject of intense investigation. Previous research hasshown that HJ containes a wide variety of chemical compounds, including flavonoids and their corresponding glucosides [6,7,8], caffeoylquinic acids [4,5], sesquiterpenes [19], triterpenes [9], and unsaturated fatty acids [20]. Among them, caffeoylquinic acids, flavonoids, and their corresponding glycosides were considered to be the main bioactive ingredients. As such, it is imperative that efforts are exerted to develop more new bioactive compounds from this flower. We herewith report the isolation and elucidation of two new caffeoylquinic acid derivatives (**1**, **2**), a new flavanone glycoside (**3**), and six reported flavanones (**4**–**9**) (Figure 1). The structures of the new compounds were identified by analyzing spectroscopic data (1D and 2D NMR, MS, IR, ECD, ORD, and UV). The isolated compounds **1**–**8** were also examined for their neuroprotective and hepatoprotective properties. The isolation, structure determination, and bioactivity of these compounds are described in this paper.

## 2. Results and Discussion

### 2.1. Structural Elucidation

Here, an 80% ethanol extract of *C. morifolium* flowers *cv.* HJ was fractionated sequentially with petroleum ether, EtOAc, and n-butanol, and the n-butanol fraction was successively subjected to column chromatography with HP-20 and SP-700 macroporous absorption resins, Sephadex LH-20, and purified by semi-preparative RP–HPLC to afford three new compounds (**1**–**3**) and six previously known compounds (**4–9**) (Figure 1). By comparing their NMR and MS data with values reported in the literature, the known isolates were identified as eriodictyol (**4**) [21], eriodictyol 7-*O*-β-d-glucopyranoside (**5**) [22], eriodictyol 7-*O*-β-d-glucuronide (**6**) [22], eriodictyol 7-*O*-β-d-rutinoside (**7**) [23], hesperetin 7-*O*-β-d-glucuronide (**8**) [22], dehydrokaempferide (**9**) [24].

**Figure 2:**
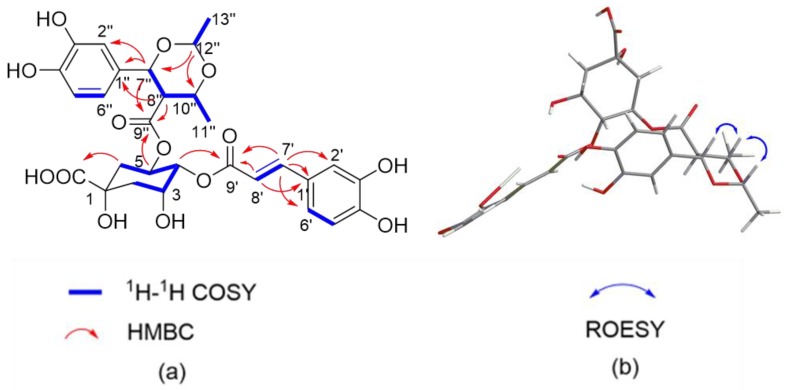
(**a**) ^1^H-^1^H COSY and key HMBC correlations of compound **1**; (**b**) selected ROESY correlations of compound **1**.

**Figure 3:**
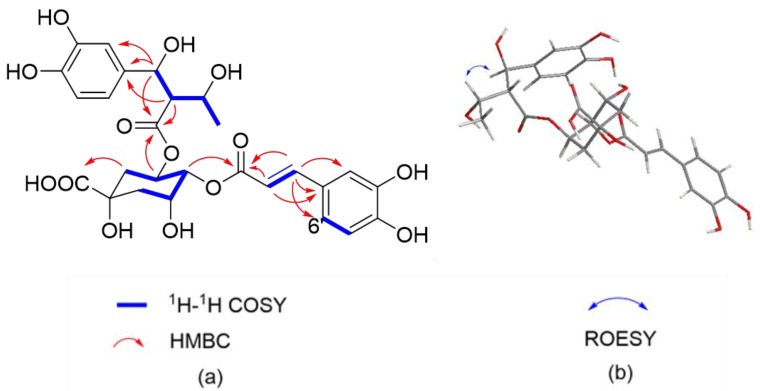
(**a**) ^1^H-^1^H COSY and key HMBC correlations of Compound **1**; (**b**) selected ROESY correlations of compound.

Compound **1,** a white, amorphous power, possesses a molecular formula of C_29_H_32_O_14_, which was determined by HRESIMS (*m*/*z* 627.1704 [M + Na]^+^, calculated for C_29_H_32_NaO_14_, 627.1684) and ^13^C-NMR data, corresponding to 14 degrees of unsaturation. The IR spectrum displayed characteristic absorptions of hydroxy (3365 cm^−1^), carbonyl (1711 cm^−1^), and aromatic ring (1522 and 1445 cm^−1^) groups. The ^1^H- NMR data (Table 1) of **1** showed signals readily corresponding to two sets of ABX systems at *δ*_H_ 7.03 (1H, d, *J* = 2.0 Hz, H-2′′), 6.78 (1H, d, *J* = 8.0 Hz, H-5′), 6.94 (1H, dd, *J* = 8.0, 2.0 Hz, H-6′) and 6.76 (1H, d, *J* = 2.0 Hz, H-2′′), 6.67 (1H, d, *J* = 8.0 Hz, H-5′′), 6.58 (1H, dd, *J* = 8.0, 2.0 Hz, H-6′′), belonging to two tri-substituted benzene rings, and two typical *trans* olefinic protons at *δ*_H_ 7.58 (1H, d, *J* = 16.0 Hz, H-7′) and 6.24 (1H, d, *J* = 16.0 Hz, H-8′). The proton spin system of H-7′/H-8′ in ^1^H-^1^H COSY (Figure 2a) spectra was observed, in addition to HMBC correlations (Figure 2a) from H-7′ to C-1′, C-2′, C-6′, and C-9′ and from H-8′ to C-1′ and C-9′. Taken together, the above NMR data suggested the existence of a caffeoyl motif in the structure. A quinic acid moiety was revealed via ^1^H NMR resonances of three oxymethine protons at *δ*_H_ 5.44 (m, H-5), 4.90 (m, H-4), and 4.28 (m, H-3), together with two sets of sp^3^ methylene protons at *δ*_H_ 2.06 (m, 2H) and *δ*_H_ 1.70 (2H, m) for H_2_-2 and H_2_-6, respectively, as shown in Table 1. In combination with the HSQC correlations, these resonances corresponded to three oxygenated methine carbons at *δ*_C_ 69.4 (C-5), 76.3 (C-4), 70.3 (C-3), as well as to two sp^3^ methylene carbons at *δ*_C_ 40.2 (C-6) and 38.2 (C-2). In addition, there were an oxygenated tertiary carbon at *δ*_C_ 75.2 (C-1) and a carbonyl at *δ*_C_ 175.9 (C-7) in the ^13^C-NMR spectrum, which are characteristic of a quinic acid unit. The assignments for the quinic acid nucleus were confirmed by analysis of the ^1^H-^1^H COSY cross peaks H-2ax/H-3/H-4/H-5/H-6ax and HMBC correlation from H-2ax to C-7 (175.9). The HMBC correlation between *δ*_H_ 4.90 (H-4) and 168.2 (C-9′) suggested the caffeoyl group was esterified to 4-OH of the quinic acid. Besides the signals of a caffeoyl group and a quinic acid, additional 13 carbon resonances were observed. After analyzing the ^1^H, ^13^C, and HSQC data, the remaining signals were attributed to one carbonyl groups (*δ*_C_ 171.8), six aromatic carbons (one ABX system, *δ*_C_ 131.7, 115.2, 146.6, 146.7, 116.0, and 119.8), three oxygenated methane groups (*δ*_C_ 84.3, 75.4, 100.2), one methylene group (*δ*_C_ 57.2), and two secondary methyls (*δ*_C_ 20.3, 21.2). The assignments of substitution pattern and planar structure of compound **1** were further confirmed by analysis of the ^1^H-^1^H COSY and HMBC correlations. The ^1^H-^1^H COSY cross peaks H-7′′/H-8′′/H-10′′/ H_3_-11′′ together with the HMBC correlations from H-7′′, H-8′′, and C-9′′ established the structural fragment C-7′′–C-8′′–(C-9′′)–C-10′′−C-11′′. The ^1^H-^1^H COSY cross peaks H-12′′/H_3_-13′′ and HMBC correlations from H-12′′ to C-7′′ and C-10′′ enabled the establishment of a 2,6-dimethyl-5-carboxyl-1,3-dioxane ring. The HMBC correlations from H-7′′ to C-1′′, C-2′′, and C-6′′ and from H-8′′ to C-1′′ indicated the connectivity C-7′′–C-1′′. Moreover, the HMBC correlations from H-5 to C-9′′ confirmed C-9′′ is connected with C-3 via an ester bond. Thus, the planar structure of **1** is shown in Figure 1. The relative configuration of **1** was determined by the coupling constant and ROESY data (Figure 2b). The *J*_7′′, 8′′_ and *J*_8′′, 10′′_ values of 10.0 Hz [25] together with the ROESY correlations from H-10′′ to H-7′′ and H-12′′ suggested that H-7′′, H-10′′, and H-12′′ are *cis*-orientated. Attempts to determine the absolute configuration of compound **1** were unsuccessful due to the insufficient amount of sample. Thus, the structure of **1** was identified as (*rel*)-(7′′*R*,8′′*R*,10′′*R*,12′′*R*)-5-*O*-[4-(3, 4-dihydroxyphenyl)-2,6-dimethyl-1,3-dioxane-5-carboxyl]-4-*O*-caffeoylquinic acid. Determined by HRESIMS (*m*/*z* 601.1537 [M + Na]^+^, calculated for C_27_H_30_NaO_14_, 601.1528), compound **2**, a white, amorphous power, possesses the molecular formula of C_27_H_30_O_14_, corresponding to 13 degrees of unsaturation. The UV spectra (λmax 330, 288 nm) was highly similar to that of **1** (λmax 331, 288 nm), which indicated that compound **2** may be a caffeoylquinic acid derivative as well. The detailed analyses of 1D NMR (Table 1) and HSQC spectra suggested the presence of 3 carbonyls, 12 aromatic carbons, 2 olefinic carbons, 1 secondary methyl, 2 methylenes, 6 oxygenated methines, and 1 oxygenated quaternary carbon. Compared with those of **1**, these characteristic signals imply that one quinic moiety and one caffeoyl group exist in the structure of **2**. The assignments of substitution pattern and planar structure of compound **2** were further confirmed by analysis of the ^1^H-^1^H COSY and HMBC correlations (Figure 3a). The ^1^H-^1^H COSY cross peaks H-7′′/H-8′′/H-10′′/ H3-11′′ together with the HMBC correlations from H-7′′, H-8′′, and H-10′′ established the structural fragment C-7′′–C-8′′–(C-9′′)–C-10′′−C-11′′. The HMBC correlations from H-7′′ to C-1′′, C-2′′, and C-6′′ and from H-8′′ to C-1′′ indicated the connectivity C-7′′–C-1′′. The HMBC correlations from H-2′′ to C-7′′ and from C-4′′ and H-6′′ to C-4′′ along with that from H-5′′ to C-3′′ suggested the presence of a 1, 3, 4-trisubstituted phenyl unit. Moreover, the HMBC correlations from H-3 to C-9′′ confirmed C-9′′ is connected with C-3 via an ester bond, whereas the HMBC cross-peak of H-4 with its ester carbonyl carbon C-9′ indicated the attachment of the caffeoyl group at C-4. On the basis of these results, the planar structure of 2 was assigned as shown in Figure 1. In the ROESY spectra (Figure 3b), the correlation from H-7′′ to H-10′′, together with the coupling constant of H-7′′ and H-8′′, confirmed that H-7′′ and H-8′′ are *trans*-orientated, and H-7′′ and H-10′′ are cofacial. Consequently, **2** was established as (*rel*)-(7′′*R*,8′′*R*,10′′*R*,12′′*R*)-5-*O*-[8-(1′-hydroxyethyl)]-dihydrocaffeoyl-4-*O*-caffeoylquinic aicd.

**Figure 4:**
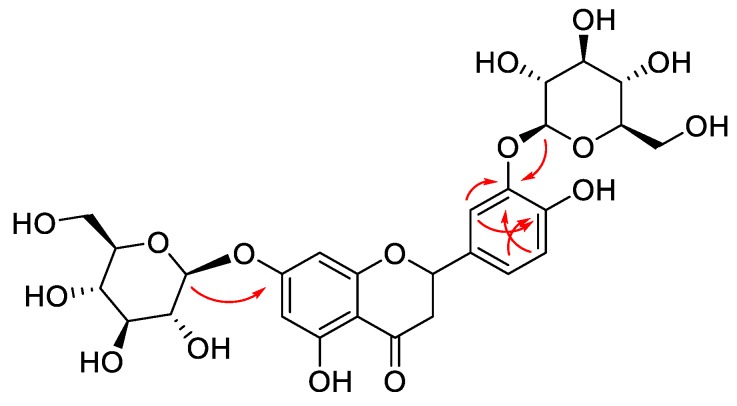
Key HMBC correlations of compound **3**.

The molecular formula of compound **3**, a white, amorphous solid, was assigned to be C_27_H_32_O_16_, as deduced from HRESIMS (*m*/*z* 635.1554 [M + H]^+^ and calculated for C_27_H_32_NaO_16_, 635.1583) and ^13^C-NMR spectra. The IR spectrum showed absorption bands at 1580, 1519, and 1441 cm^−1^ (benzyl) and strong absorption bands at 3328 and 1635 cm^−1^ (hydroxyl, carbonyl, respectively). The ^1^H-NMR data showed resonances at *δ*_H_ 5.50 (1H, m, H-2), 3.32 (1H, m, H-3), and 2.75 (1H, dd, *J* = 17.0, 2.0 Hz, H-3) (Table 2). These evidence, together with characteristic UV absorption bands at 283, 333 nm, suggested the presence of a flavanone skeleton [26]. The ^1^H-NMR data showed one ABX spin system aromatic proton signals at *δ*_H_ 7.38 (1H, d, *J* = 2.0 Hz, H-2′), 7.03 (1H, dd, *J* = 8.0, 2.0 Hz, H-6′), 6.85 (1H, d, *J* = 2.0 Hz, H-5′), attributed to the B ring of the flavanone skeleton, two aromatic doublets at *δ*_H_ 6.17 (1H, d, *J* = 2.0 Hz, H-8) and 6.14 (1H, d, *J* = 2.0 Hz, H-6), assignable to the A ring, as well as well as two phenolic hydroxyl groups at *δ*_H_ 12.06 and 8.84. In addition, protons of two *β*-glucose moieties, for which the anomeric protons resonated at *δ*_H_ 4.97 (1H, d, *J* = 7.5 Hz, H-1′′), 4.73 (1H, d, *J* = 7.0 Hz, H-1′′′), respectively, were found in the high-field region. The ^13^C-NMR spectrum exhibited 27 carbon signals, corresponding to a flavanone skeleton bearing two hydroxyl units and two sugar moieties. The locations of two glucose units were determined at C-7 and C-3′ on the basis of the cross-peak from the anomeric protons H-1″ to C-7, and H-1″′ to C-3′ in the HMBC spectra (Figure 4). The coupling constant (*J* = 7.5 Hz, H-1′′ and H-1′′′) of two anomeric protons indicated two β-glycosidic linkages. The d-configuration of the glucose moieties was determined via GC analysis of its chiral derivatives after acid hydrolysis. Moreover, the stereochemistry at C-2 was established as *S* due to the presence of a negative Cotton effect at 287 nm and a positive Cotton effect at 342 nm in the circular dichroism (CD) spectrum (see Supplementary Materials) [27]. Accordingly, the structure of **3** was identified as (2*S*)-eriodictyol 7,3′-di-*O*-β-d-glucopyranoside.

### 2.2. Biological Activities

#### 2.2.1. Hepatoprotective Activity 

**Figure 5:**
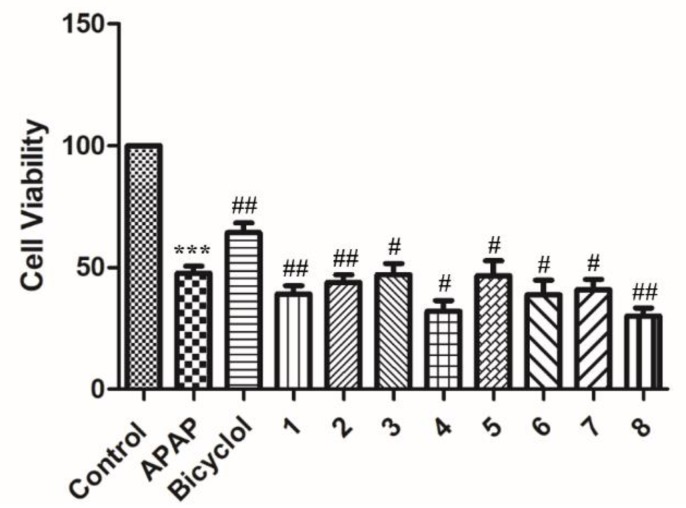
Hepatoprotective effects of compounds **1**–**8** against *N*-acetyl-*p*-aminophenol (APAP)-induced HepG2 cell injury.

Compounds **1**–**8** were tested for their hepatoprotective activities against *N*-acetyl-*p*-aminophenol (APAP)-induced injury in HepG2 cells using bicyclol as a positive control in the MTT method. All compounds were inactive at a concentration of 10 μM (Figure 5).

#### 2.2.2. Neuroprotective Activity 

**Figure 6:**
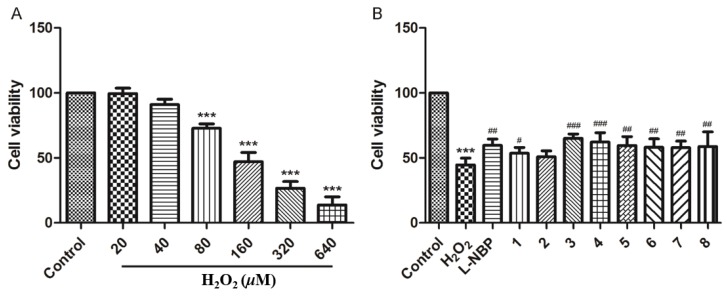
(**A**) Toxicity of different H_2_O_2_ concentrations in SH-SY5Y cells; (**B**) neuroprotective effect of compounds **1**–**8** against H_2_O_2_-induced toxicity in SH-SY5Y cells.

The isolated compounds **1**–**8** were tested for their neuroprotective effect against H_2_O_2_-induced cell toxicity in SH-SY5Y cells. Compounds **3** and **4** exhibited a moderate neuroprotective effect at a concentration of 10 μM against SH-SY5Y cell damage with cell viability of 65.08% and 62.24%, respectively, compared with that measured in the presence of l-NBP (l-3-n-butylphthalide) corresponding to 59.74%. Other compounds displayed mild activities, ranging from 57.19% to 59.57% in cell viability at 10 μM (Figure 6).

## 3. Materials and Methods

### 3.1. General Experimental Procedures

Data of optical rotations, UV, and ECD spectra were obtained using Jasco P2000, JASCO V-650, and Jasco J-815 spectrophotometers (Jasco Corporation, Tokyo, Japan), respectively. IR spectra were measured with a Nicolet 5700 spectrometer (Thermo Nicolet Corporation, Madison, SD, USA). GC was performed with an Agilent 7890A instrument (Agilent Technologies, Waldbronn, Germany). The 1D- and 2D-NMR spectra were obtained at 500 or 600 MHz for ^1^H and at 125 or 150 MHz for ^13^C, using Bruker 600 and 500 MHz spectrometers (Bruker Corporation, Karlsruhe, Germany). HRESIMS data were acquired with an Agilent 1100 series LC/MSD ion trap mass spectrometer (Agilent Technologies, Waldbronn, Germany). Column chromatography was performed using macroporous resin (Diaion HP-20 and SP-700, from Mitsubishi Chemical Corp., Tokyo, Japan and Sephadex LH-20 columns Pharmacia Fine Chemicals, Uppsala, Sweden. Preparative HPLC was carried out with a Shimadzu LC-6AD instrument with an SPD-20A detector Shimadzu Corp., Tokyo, Japan, using a YMC-Pack ODS-A column (250 mm × 20 mm, 5 μm; YMC Corp., Kyoto, Japan). HPLC-DAD analysis was performed using an Agilent 1200 series system (Agilent Technologies, Waldbronn, Germany) with an Apollo C18 column (250 mm × 4.6 mm, 5 μm; Alltech Corp., Lexington, KY, USA).

### 3.2. Plant Materials

The dried flower of *C. morifolium* Ramat. was collected in Tongxiang, Zhejiang province, China, in September 2014. The plant was identified by Professor Lin Ma Institute of Materia Medica, Chinese Academy of Medical Sciences, Beijing.

### 3.3. Extraction and Isolation

Dried flowers of *C. morifolium* Ramat. (100 kg) were extracted three times with 80% EtOH (3 × 150 L) under reflux for 3 h. The EtOH solution was evaporated under reduced pressure, and then the dark brown residue (6.4 kg) was suspended in H_2_O (10 L) and fractionated sequentially using petroleum ether (5 × 10 L), EtOAc (5 × 10 L), and n-BuOH (6 × 10 L). The n-BuOH-soluble fraction (2355 g) was subjected to chromatography on an HP-20 macroporous absorption resin column (200 cm × 15 cm i.d.) and eluted successively with 0, 15, 30, 50, 75, and 95% ethanol (50 L each). The 30% ethanol solution (480 g) was fractionated by column chromatography (200 cm × 10 cm i.d.) on the adsorptive macroporous resin SP-700, eluting with 10, 15, 20, 25, 30, 50, and 95% EtOH (30 L each) to afford seven fractions (fractions A–G).

Fraction D (80 g) was separated on a Sephadex LH-20 column (120 cm × 8 cm i.d.), eluting with a MeOH/H_2_O mixture (0–100%, with 10% stepwise increase of MeOH, 10 L each) to give fractions D1–D10. Fraction D5 was further chromatographed on a Sephadex LH-20 column (150 cm × 5 cm i.d.) with MeOH/H_2_O (0, 10, 20, 30, 40, 50, 60, 70, 85, and 100%, *v*/*v*, 2 L each) to generate fractions D5-1–D5-23. All of these sub-fractions were further chromatographed on Sephadex LH-20 columns and purified by semi-preparative HPLC. Fraction D5-7 afforded compounds **1** (2 mg) and **2** (5 mg), using MeOH/H_2_O/HOAc (30:70:0.1, *v*/*v*) as the mobile phase at 3 mL/min. Fraction D7 was fractionated by semi-preparative HPLC using MeOH/H_2_O/HOAc (40:60:0.1, *v*/*v*) at 3 mL/min to produce compounds **3** (18 mg), **4** (6 mg), and **5** (13 mg). Compounds **6** (9 mg), **7** (11 mg), **8** (8 mg), and **9** (6 mg) were obtained from fraction D6 using MeOH/H_2_O/HOA_C_ (40:60:0.1, *v*/*v*) as the mobile phase at 3 mL/min.

### 3.4. Characterization 

Compound **1**: White amorphous powder; ${\left[\alpha \right]}_{D}^{20}$ +146 (c 0.1, MeOH); UV (MeOH) λmax (log ε) 330 (4.15), 288 (3.89) nm; IR (KBr) *ν*_max_ 3365, 1711, 1603, 1522, 1495, 1375, 1264, 978, 817 cm^−1^; NMR data see Table 1; HRESIMS *m*/*z* 627.1704 [M + Na]^+^ (calculated for 627.1684, C_29_H_32_NaO_14_).

Compound **2**: White amorphous powder; ${\left[\alpha \right]}_{D}^{20}$ +125 (c 0.1, MeOH); UV (MeOH) λmax (log ε) 331 (3.45), 288 (3.19) nm; IR (KBr) *ν*_max_ 3378, 1711, 1605, 1523, 1449, 1377, 1282, 1077, 981, 818 cm^−1^; NMR data see Table 1; HRESIMS *m*/*z* 601.1537 [M + Na]^+^ (calculated for 601.1528, C_27_H_30_NaO_14_).

Compound **3**: White amorphous powder; ${\left[\alpha \right]}_{D}^{20}$ ‒116 (c 0.1, MeOH); UV (MeOH) λmax (log ε) 333 (sh), 283 (3.79) nm; IR (KBr) *ν*_max_ 3329, 2905, 1648, 1580, 1519, 1441, 1400, 1371, 1297, 1203, 1084, 973, 907cm^−1^; NMR data see Table 1; HRESIMS *m*/*z* 635.1554 [M + Na]^+^ (calculated for 635.1583, C_27_H_32_NaO_16_).

### 3.5. Hydrolysis of Compound 3 and GC Analysis

Compounds (4 mg) was hydrolyzed with 2 M HCl (3 mL) at 80 °C for 5 h. The sugars gained from the hydrolysates were analyzed by GC on the basis of the reported method [28].

### 3.6. Hepatoprotective Activity Assay

HepG2 cells were cultured in DMEM with 10% FCS, penicillin (100 U/mL), and streptomycin (100 μg/mL) at 37 °C (5% CO_2_, 100% relative humidity). These cells were digested using 0.25% trypsin and then seeded into 96-well plates. After incubation for 24 h, the cells were treated with the isolated compounds (10 μM) and APAP (8 mM) and incubated for 48 h. A volume of 100 μL of MTT solution (0.5 mg/mL) was then added into each well. After incubation for 4 h at 37 ℃, the cells were finally lysed with 150 μL DMSO. Finally, the absorbance was measured at 570 nm with a microplate reader, using bicyclol as the positive control. The results were expressed as percentage of cell viability (%), hypothesizing the viability of control cells as 100%.

### 3.7. Neuroprotective Activity Assay

Human neuroblastoma SH-SY5Y cells were maintained in DMEM supplemented with 10% FBS, 100 U/mL penicillin, and 100 µg/mL streptomycin at 37 ℃ under a water-saturated atmosphere of 5% CO_2_. The cells were seeded in 96-well culture plates (1 × 10^4^ cells/well) in 100 μL for 18 h, then incubated with the isolated compounds (10 μL, 10 μM) for 4 h. In order to induce an oxidative stress, 100 μL freshly prepared culture medium with 200 μM H_2_O_2_ (IC_50_ = 128.5 μM) was added to the cells and incubated with the compounds at 37 °C for 24 h. Then, 10 μL MTT solution (5 mg/mL) was added into each well. After incubation for 4 h at 37 °C, the cells were finally lysed with 150 μL DMSO. The absorbance was measured at 570 nm, using l-NBP (l-3-n-butylphthalide) as the positive control. The results were expressed as percentage of cell viability (%).

## 4. Conclusions

In summary, two new caffeoylquinic acid derivatives (**1,2**), a new flavanone glycoside (**3**), and six reported flavanone glycosides (**4**–**9**), were isolated and characterized from the dried flowering head of *C. morifolium* Ramat. The in vitro evaluation of neuroprotective activity suggested that compounds **3** and **4** could improve cell viability at a concentration of 10 μM. This paper not only enriches the chemical diversity of *C. morifolium* compounds but also provides useful clues on the neuroprotective agents of *C. morifolium* Ramat cv. “HJ”, which will contribute to the development and application of this functional food. Further investigations should be implemented to examine the neuroprotective activity in vivo and the required isolates’ concentrations and provide real biological significance in the future.

## Figures and Tables

**Table 1:** NMR spectroscopic data for compounds **1**, **2** in methanol-*d*_4_ (*δ* in ppm, *J* in Hz).

	1 ^a^		2 ^b^	
Position	*δ*_H_	*δ*_C_	*δ*_H_	*δ*_C_
1		75.2		75.1
2	2.06, m	38.2	2.01, m 2.19, m	38.4
3	4.28, m	70.3	4.24, m	70.1
4	4.90, m	76.3	5.01, dd (3.0, 10.0)	75.7
5	5.44, m	69.4	5.29, m	69.8
6	1.70, m	40.2	1.89, m	39.9
7		175.9		175.2
1′		127.7		127.7
2′	7.03, d (2.0)	115.1	7.07, d (2.0)	115.2
3′		146.8		146.8
4′		149.8		149.7
5′	6.78, d (8.0)	116.5	6.78, d (8.0)	115.9
6′	6.94, dd (2.0, 8.0)	123.1	6.97, dd (2.0, 8.0)	123.2
7′	7.58, d (16.0)	147.8	7.63, d (16.0)	147.6
8′	6.24, d (16.0)	114.9	6.27, d (16.0)	114.9
9′		168.2		168.4
1′′		131.7		134.3
2′′	6.76, d (2.0)	115.2	6.66, d (2.0)	115.4
3′′		146.6		146.2
4′′		146.7		146.5
5′′	6.67, d (8.0)	116.0	6.66, d (8.0)	116.5
6′′	6.58, dd (2.0, 8.0)	119.8	6.81, dd (2.0, 8.0)	120.2
7′′	4.56, d (10.0)	81.8	4.77, d (10.0)	76.7
8′′	2.38, dd (10.0, 10.0)	57.2	2.72, dd (8.5, 10.0)	62.4
9′′		171.8		171.2
10′′	3.93, dq (6.0, 10.0)	75.4	4.14, dq (6.0, 8.5)	70.3
11′′	1.10, d (6.0)	20.3	1.14, d (6.0)	21.7
12′′	4.90, m	100.2		
13′′	1.29, d (5.0)	21.2		

^a 1^H and ^13^C-NMR were measured at 500 and 150 MHz. ^b 1^H and ^13^C-NMR were measured at 500 and 125 MHz.

**Table 2:** NMR spectroscopic data for compound **3** in DMSO-*d*_6_ (500 MHz for ^1^H NMR, 125 MHz for ^13^C NMR, *δ* in ppm, *J* in Hz).

	3			3	
Position	*δ*_H_	*δ*_C_	Position	*δ*_H_	*δ*_C_
2	5.50, m	78.8	6′	7.03, dd (2.0, 8.0)	121.9
3	3.32, m 2.75, dd (2.0, 17.0)	42.0	1′′	4.97, d (7.5)	99.6
4		197.3	2′′	3.2–3.4, m	73.1
5		162.8	3′′	3.2–3.4, m	76.1
6	6.14, d (2.0)	96.6	4′′	3.2–3.4, m	69.5
7		165.4	5′′	3.2–3.4, m	77.1
8	6.17, d (2.0)	95.5	6′′	3.67, m 3.47, m	60.6
9		163.0	1′′′	4.73, d (7.0)	101.9
10		103.3	2′′′	3.2–3.4, m	73.4
1′		129.3	3′′′	3.2–3.4, m	76.4
2′	7.38, d (2.0)	115.4	4′′′	3.2–3.4, m	69.9
3′		145.2	5′′′	3.2–3.4, m	77.3
4′		147.3	6′′′	3.67, m 3.47, m	60.8
5′	6.85, d (8.0)	115.8			

## Supplementary Materials

NMR, HR-ESI-MS, IR, UV, and CD spectra of the new compounds (**1**–**3**) in this article are available in the online version.

## References

[1] Chinese Pharmacopeia Commission (2010). Pharmacopeia of the People′s Republic of China.

[2] Ma R.L., Huang C.L., Zhang X.H., Wu Z.Y. (2009). Advancement on the study of tea *chrysanthemum*. North. Hortic..

[3] Peng Y.R., Shi L., Luo Y.H., Ding Y.F. (2006). Protective effect of total flavones from *chrysanthemum* on isoprenaline-induced myocardial ischemia in rats. Lishizhen Med. Mater. Med. Res..

[4] Kim H.J., Lee Y.S. (2005). Identification of new dicaffeoylquinic acids from *Chrysanthemum morifolium* and their antioxidant activities. Planta Med..

[5] Duh P.D., Yen G.C. (1997). Antioxidative activity of three herbal water extracts. Food Chem..

[6] He D.X., Ru X.C., Wen L., Wen Y.C., Jiang H.D., Bruce I.C., Jin J., Ma X., Xia Q. (2012). Total flavonoids of *Flos Chrysanthemi* protectarterial endothelial cells against oxidative stress. J. Ethnopharmacol..

[7] Lee J.S., Kim H.J., Lee Y.S. (2003). A new anti-HIV flavonoid glucuronide from *Chrysanthemum morifolium*. Planta Med..

[8] Hu C.Q., Chen K., Shi Q., Kilkuskie R.E., Cheng Y.C., Lee K.H. (1994). Anti-AIDS agents, 10. Acacetin-7-*O*-*β*-D-galactopyranoside, an anti HIV principle from *Chrysanthemum morifolium* and a structure- activity correlation with some related flavonoids. J. Nat. Prod..

[9] Ukiya M., Akihisa T., Yasukawa K., Kasahara Y., Kimura Y., Koike K., Nikaido T., Takido M. (2001). Constituents of compositae plants. 2. Triterpene diol, triols, and their 3-*O*-fatty acid esters from edible *chrysanthemum* flower extract and their anti-inflammatory effects. J. Agric. Food Chem..

[10] Jiang H.D., Wang L.F., Zhou X.M., Xia Q. (2005). Vasorelaxant effects and underlying mechanism of EtOAc extract from *Chrysanthemum morifolium* in rat thoracic aorta. Chin. J. Pathophysiol..

[11] Kim I.S., Koppula S., Park P.J., Kim E.H., Kim C.J., Choi W.S., Lee K.H., Choi D.K. (2009). *Chrysanthemum morifolium* Ramat (CM) extract protects human neuroblastoma SH-SY5Y cells against MPP^+^-induced cytotoxicity. J. Ethnopharmacol..

[12] Ukiya M., Akihisa T., Tokuda H., Suzuki H., Mukainaka T., Ichiishi E., Yasukawa K., Kasahara Y., Nishino H. (2002). Constituents of Compositae plants III. Anti-tumor promoting effects and cytotoxic activity against human cancer cell lines of triterpene diols and triols from edible *chrysanthemum* flowers. Cancer Lett..

[13] Xie Y.Y., Yuan D., Yang J.Y., Wang L.H., Wu C.F. (2009). Cytotoxic activity of flavonoids from *Flos Chrysanthemum* on human colon cancer Colon 205 cells. J. Asian Nat. Prod. Res..

[14] Kang W.Y., Huang X., Lian T.T., Xu Q.T. (2012). Protective Effect of *Dendranthema morifolium* on CCl_4_- induced Liver Injury in Mice. Nat. Prod. Res. Dev..

[15] Wang P., Pan X., Chen G. (2012). Increased exposure of vitamin A by *Chrysanthemum morifolium* Ramat extract in rat was not via induction of CYP1A1, CYP1A2 and CYP2B1. J. Food Sci..

[16] Matsuda H., Morikawa T., Toguchida I., Yoshikawa M. (2002). Structural requirements of flavonoids and related compounds for aldose reductase inhibitory activity. Chem. Pharm. Bull..

[17] Terashima S., Shimizu M., Horie S., Morita N. (1991). Studies on aldose reductase inhibitors from natural products. IV. Constituents and aldose reductase inhibitory effect of *Chrysanthemum morifolium*, Bixa orellana and Ipomoea batatus. Chem. Pharm. Bull..

[18] Miyazawa M., Hisama M. (2003). Antimutagenic activity of flavonoids from *Chrysanthemum morifolium*. Biosci. Biotechnol. Biochem..

[19] Hu L.H., Chen Z.L. (1997). Sesquiterpenoid alcohols from *Chrysanthemum morifolium*. Phytochemistry.

[20] Tsao R., Attygalle A.B., Schroeder F.C., Marvin C.H., McGarvey B.D. (2003). Isobutylamides of unsaturated fatty acids from *Chrysanthemum morifolium* associated with host-plant resistance against the western Flower Thrips. J. Nat. Prod..

[21] Wang X.G., Shen L.T., Zeng Y.Y., Tian Y.Q., Xu H.H. (2010). Flavonoids from *Ficus sarmentosa* var. *henryi*. Chin. Tradit. Herb. Drugs.

[22] Sun Y., Ma X.B., Liu J.X. (2012). Compounds from fraction with cardiovascular activity of *Chrysanthemum indicum*. China J. Chin. Mater. Med..

[23] Zhou D.N., Ruan J.L., Cai Y.L. (2008). Flavonoids from Aerial Parts of *Arachniodes exilis*. Chin. Pharm. J..

[24] Shang H.Q., Qin M.J., Wu J.R. (2007). Constituents of Rhizomes of *Iris tectorum*. Chin. J. Nat. Med..

[25] Abate A., Brenna E., Costantini A., Fuganti C., Gatti F.G., Malpezzi L., Serra S. (2006). Enzymatic approach to enantiomerically pure 5-alken-2,4-diols and 4-hydrocy-5-alken-2-ones: Application to the synthesis of chiral synthons. J. Org. Chem..

[26] Lin J.H., Chiou Y.N., Lin Y.L. (2002). Phenolic Glycosides from *Viscum angulatum*. J. Nat. Prod..

[27] Mizuno M., Kato M., Iinuma M., Tanaka T., Kimura A., Ohashi H., Sakai H. (1987). Acylated luteolin glucosides from *Salix gilgiana*. Phytochemistry.

[28] Yang Y.N., Huang X.Y., Feng Z.M., Jiang J.S., Zhang P.C. (2015). New butyrolactone type lignans from Arctii Fructus and their anti-inflammatory activities. J. Agric. Food Chem..

