# Supplementary material for: New Caffeoylquinic Acid Derivatives and Flavanone Glycoside from the Flowers of Chrysanthemum morifolium and Their Bioactivities
Source: Molecules. 2019 Feb 28;24(5):850. doi: 10.3390/molecules24050850 (PMC6429231; doi:10.3390/molecules24050850)
Supplement: Supplementary file 1 [file molecules-24-00850-s001.pdf]

Supplementary Materials

New Caffeoylquinic Acid Derivatives and Flavanone Glycosides from the Flowers of *Chrysanthemum morifolium* and Their Bioacivities

Peng-Fei Yang[1], Chun-Yu He[1], Duo-Bin Mao[1,*]

[1] College of Food and Biological Engineering, Zhengzhou University of Light Industry, Zhengzhou, 450002, China;

pf_yang@zzuli.edu.cn (P.-F.Y.); 349210263@qq.com (C.-Y.H.);

Correspondence: duobinmao@126.com; Tel.: +86-180-3713-7533

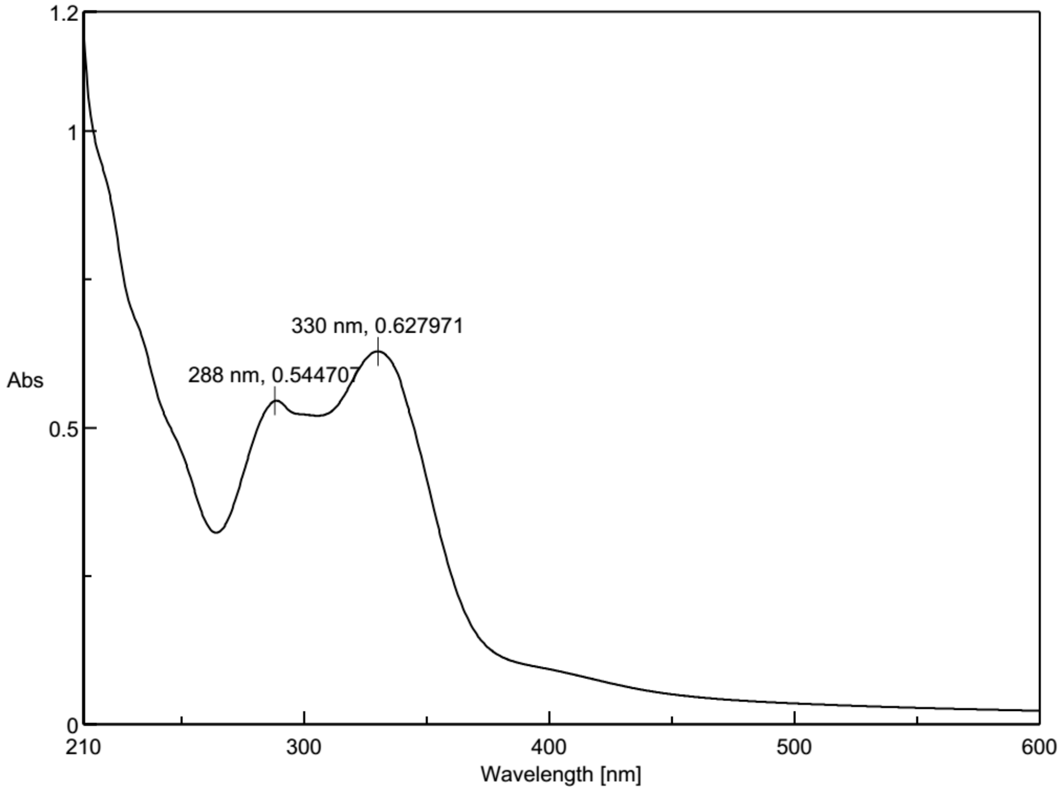

Figure S1. The UV Spectrum of compound **1** in MeOH

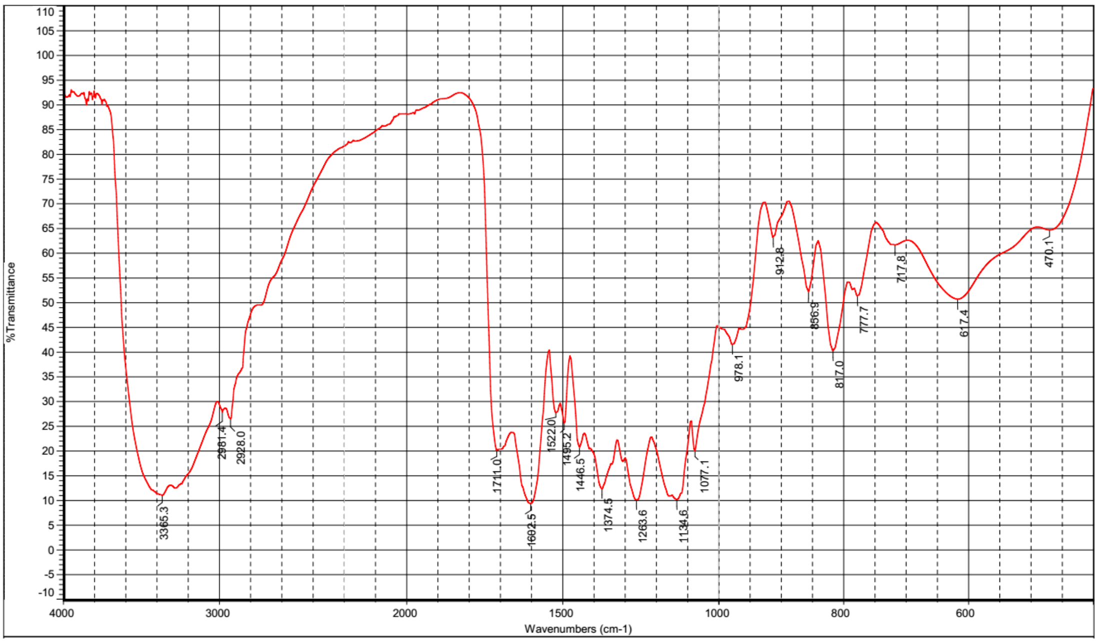

Figure S2. The IR Spectrum (KBr) of compound **1**

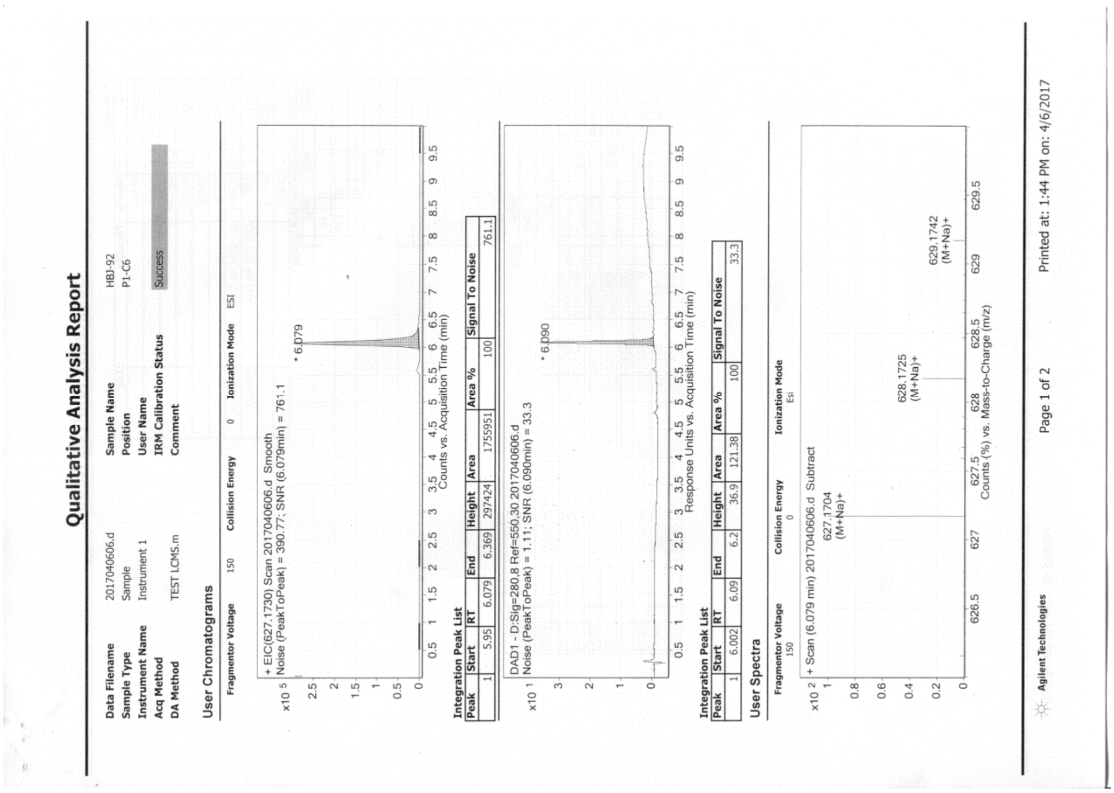

Figure S3. The HR-ESI-MS Data of compound **1**

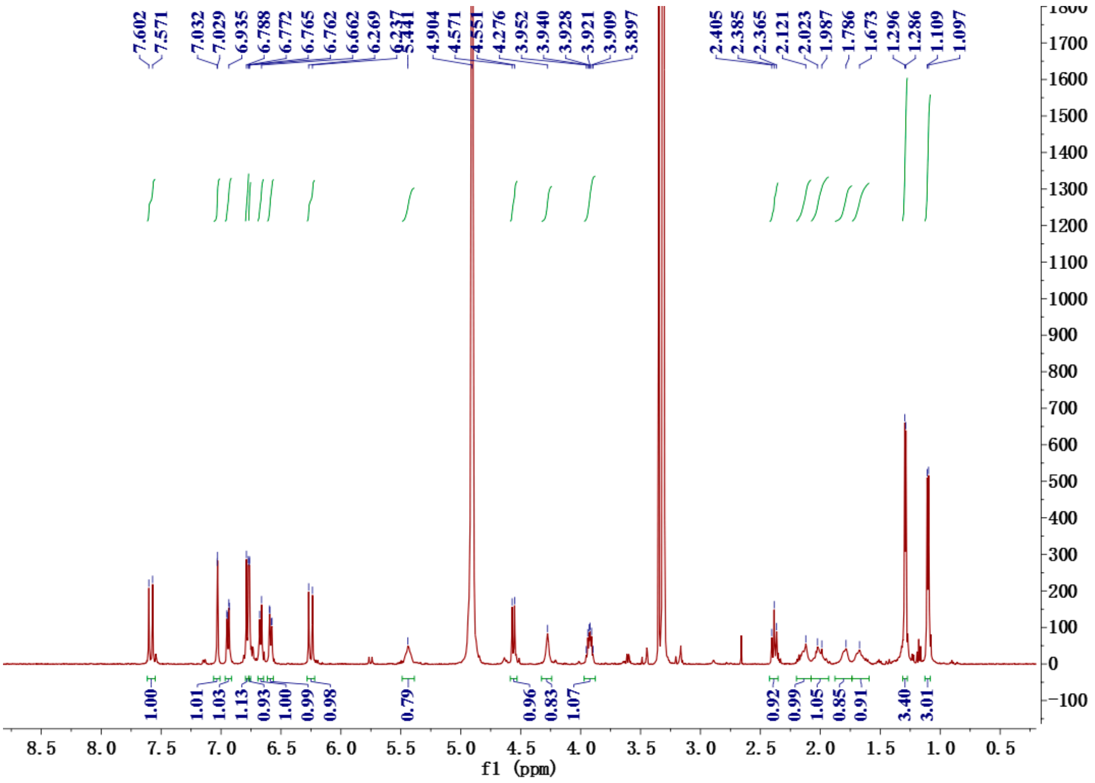

Figure S4. The ^{1}H NMR Spectrum of Compound **1** in MeOH-d_4 (500 Hz)

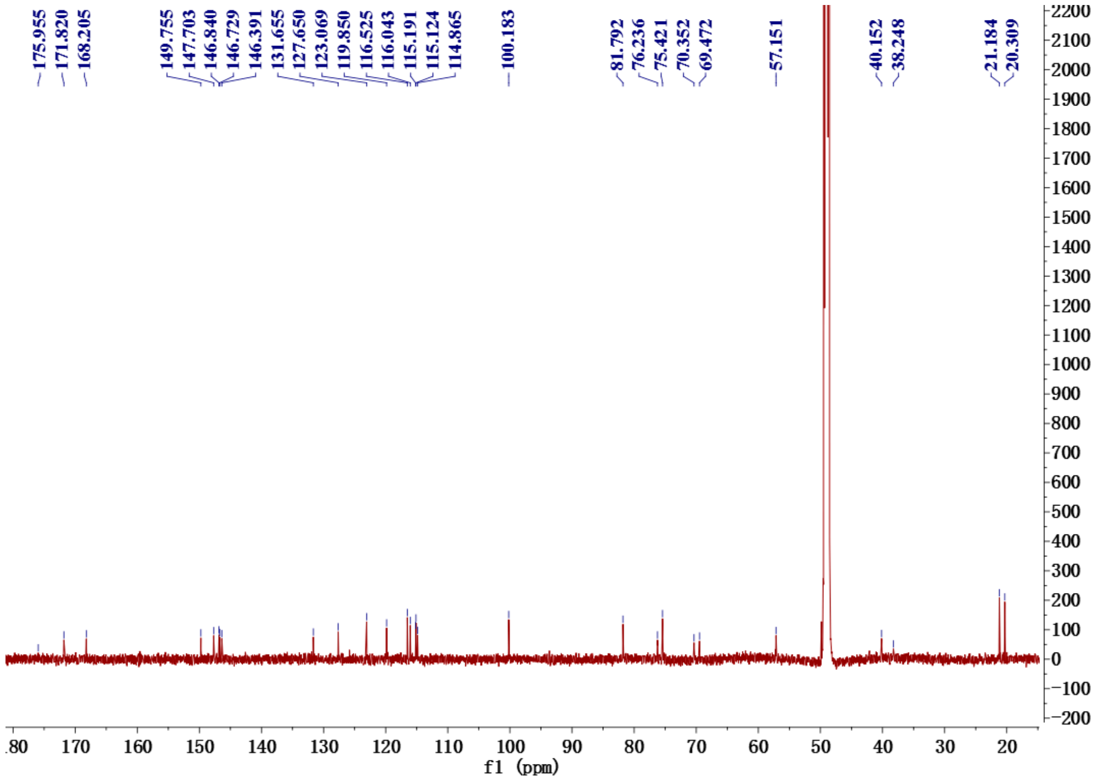

Figure S5. The ^{13}C NMR Spectrum of Compound **1** in MeOH-*d*4 (150 Hz)

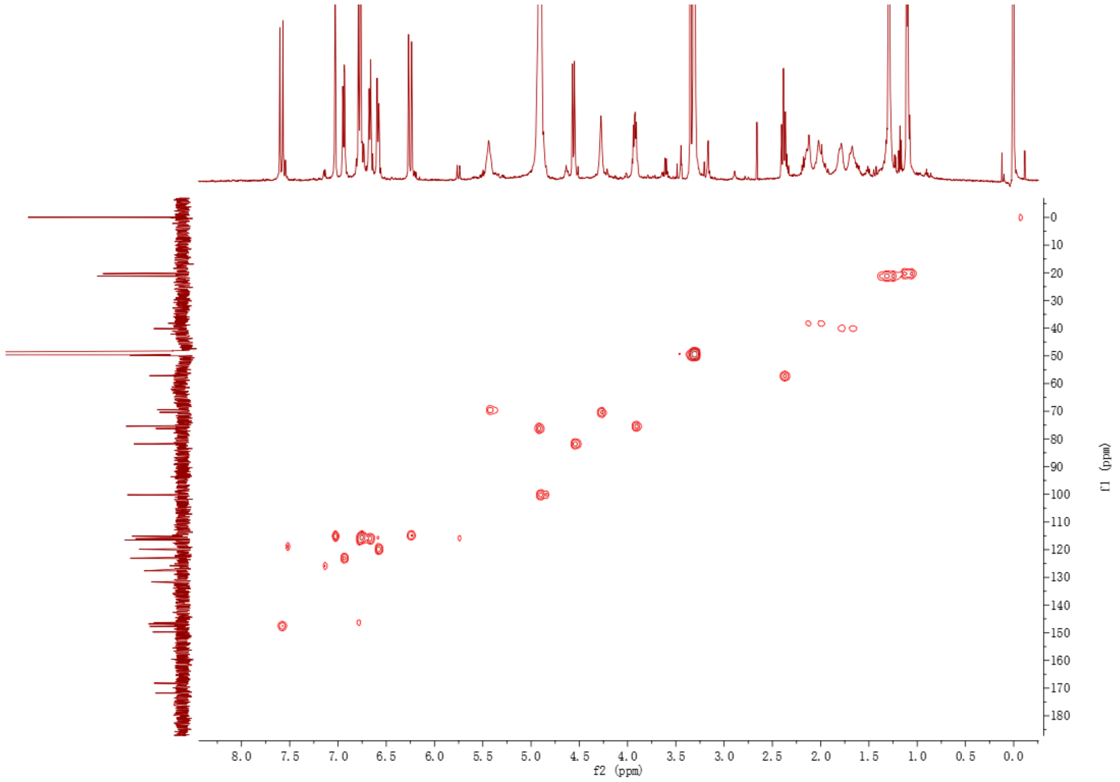

Figure S6. The HSQC Spectrum of Compound **1** in MeOH-*d*4 (600 Hz)

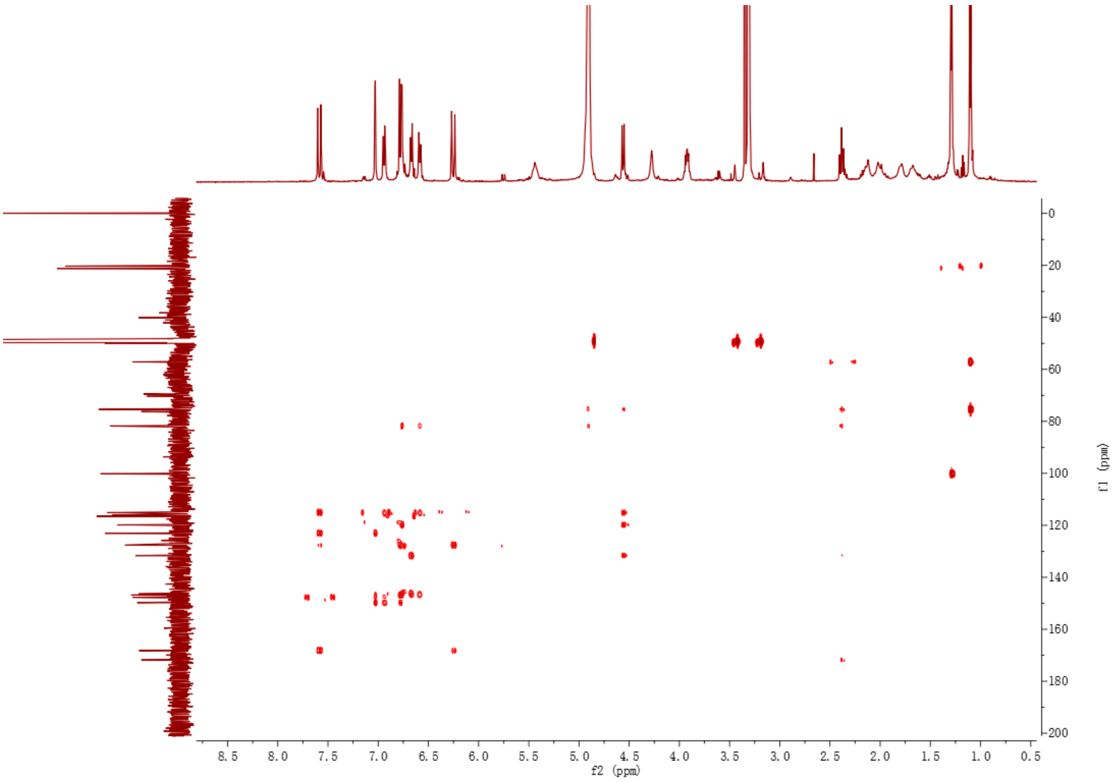

Figure S7. The HMBC Spectrum of Compound **1** in MeOH-d_4 (600 Hz)

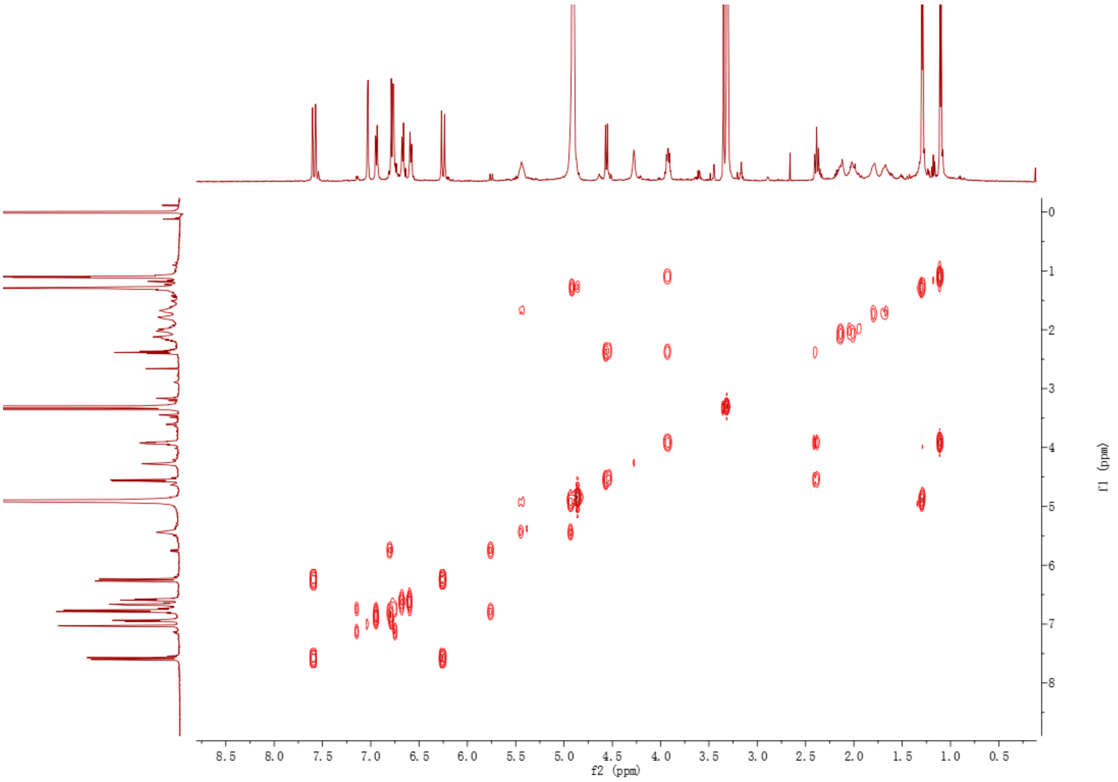

Figure S8. The ^{1}H-^{1}H COSY Spectrum of Compound **1** in MeOH-d_4 (600 Hz)

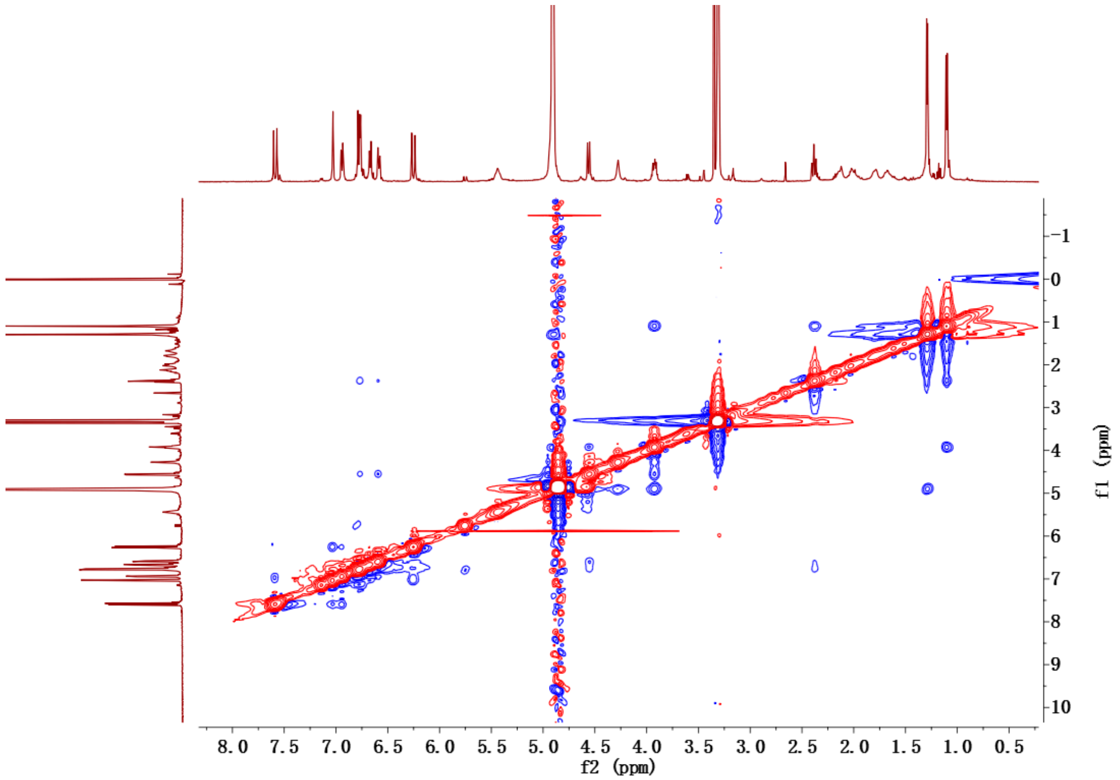

Figure S9. The ROESY Spectrum of Compound **1** in MeOH-d_4 (600 Hz)

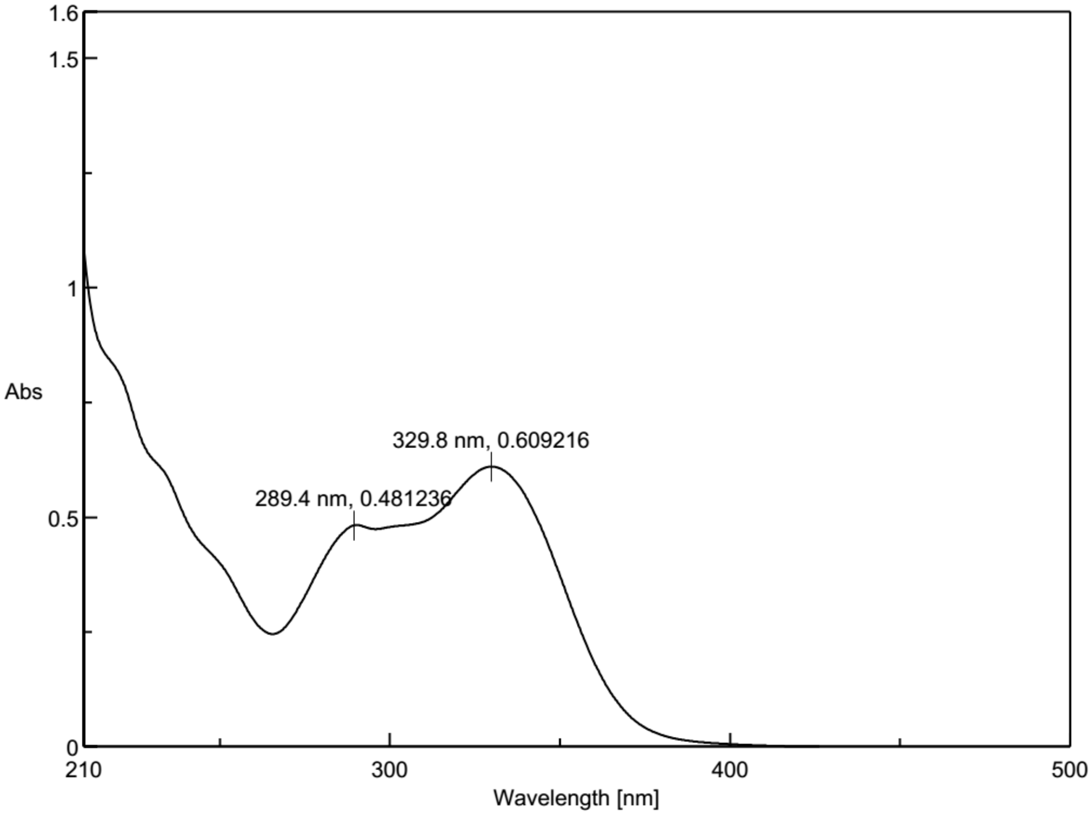

Figure S10. The UV Spectrum of compound **2** in MeOH

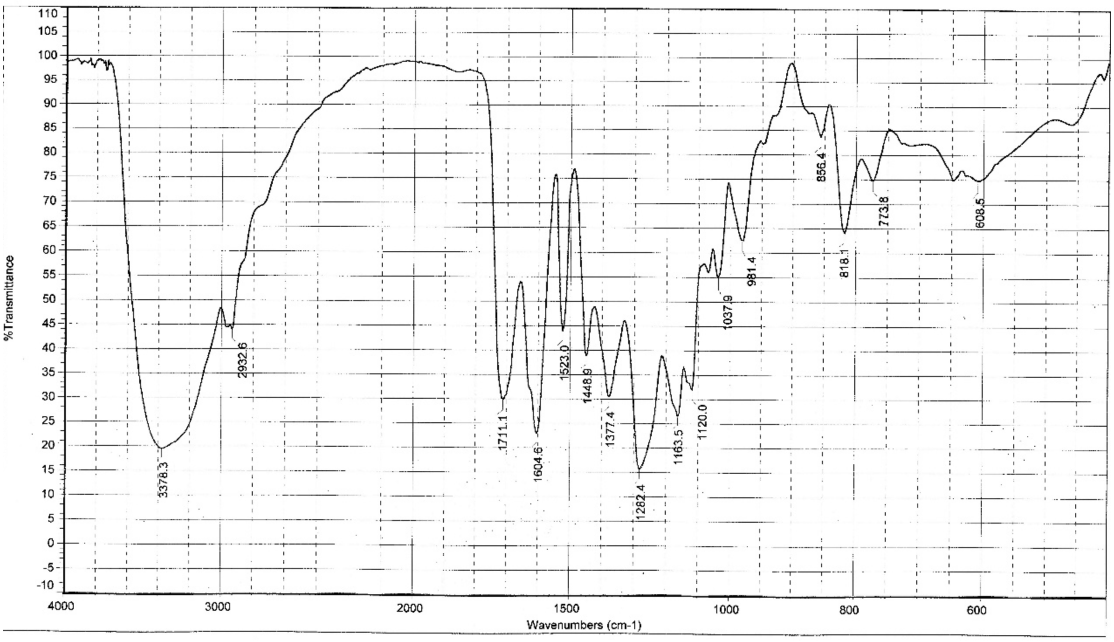

Figure S11. The IR Spectrum (KBr) of compound **2**

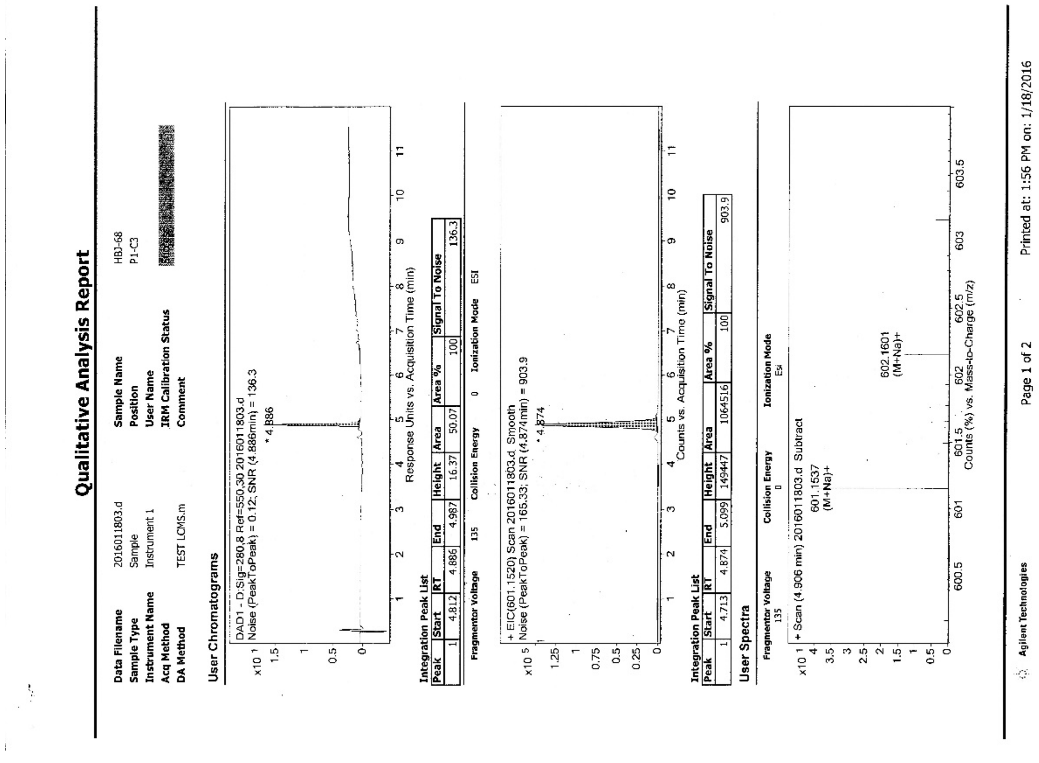

Figure S12. The HR-ESI-MS Data of compound **2**

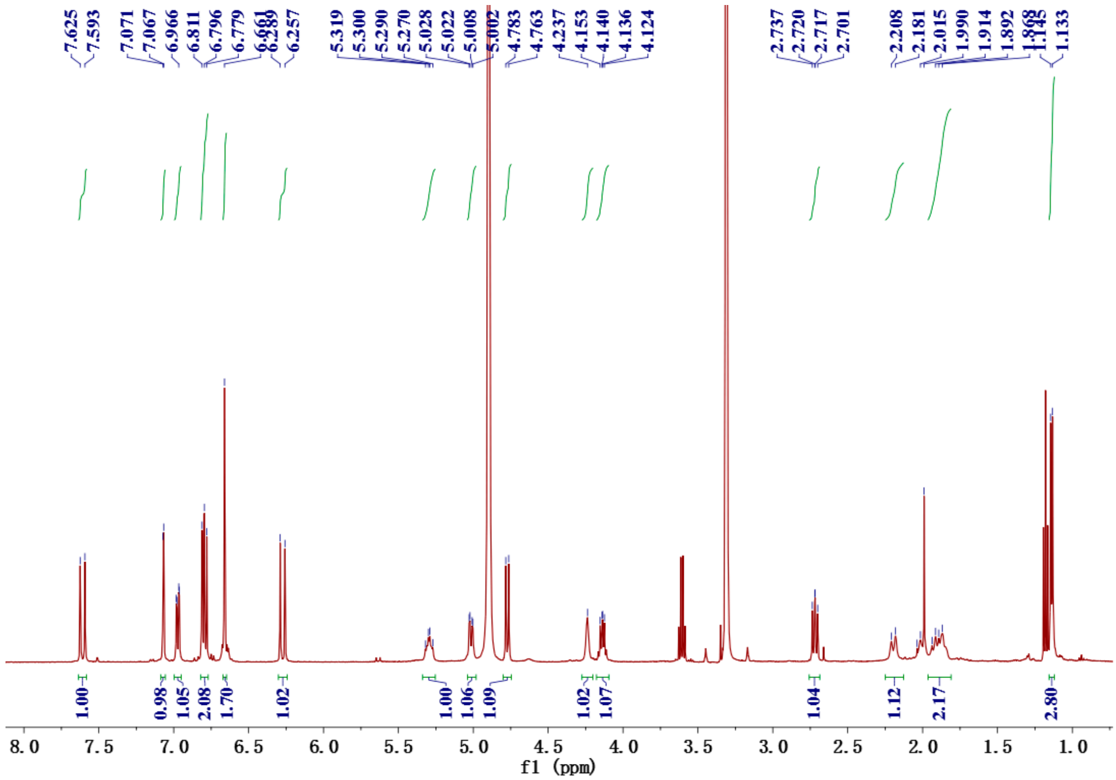

Figure S13. The ^{1}H NMR Spectrum of Compound **2** in MeOH-d_4 (500 Hz)

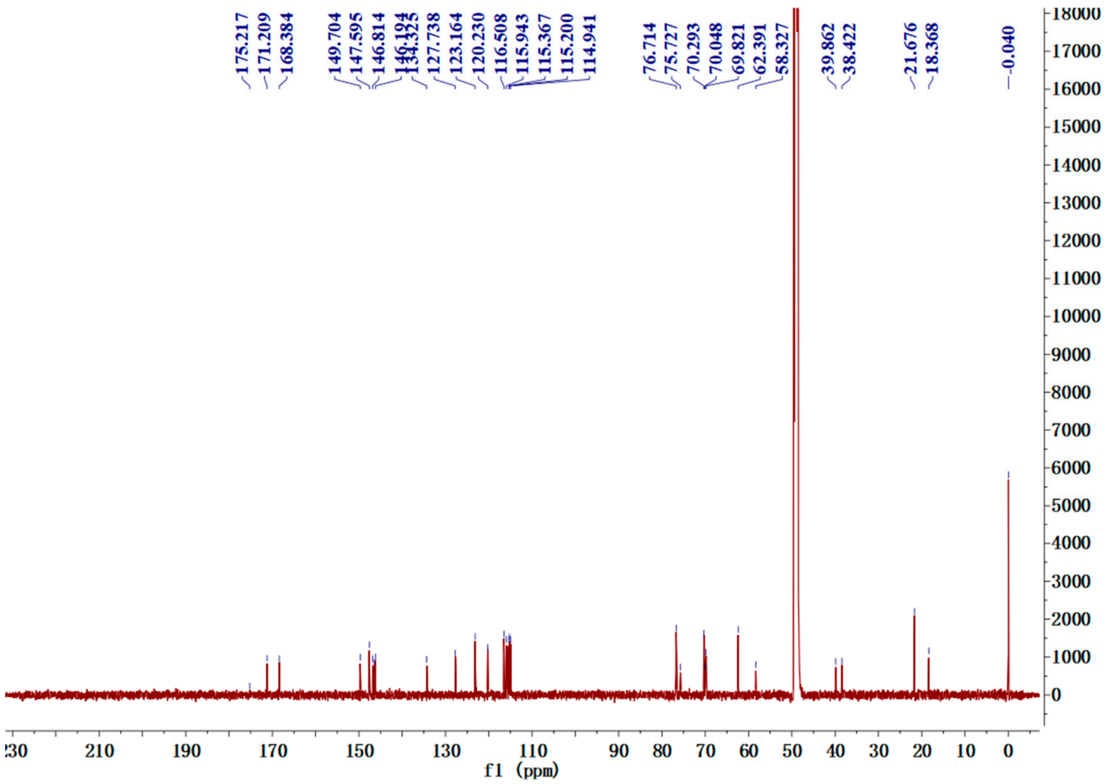

Figure S14. The ^{13}C NMR Spectrum of Compound **2** in MeOH-d_4 (500 Hz)

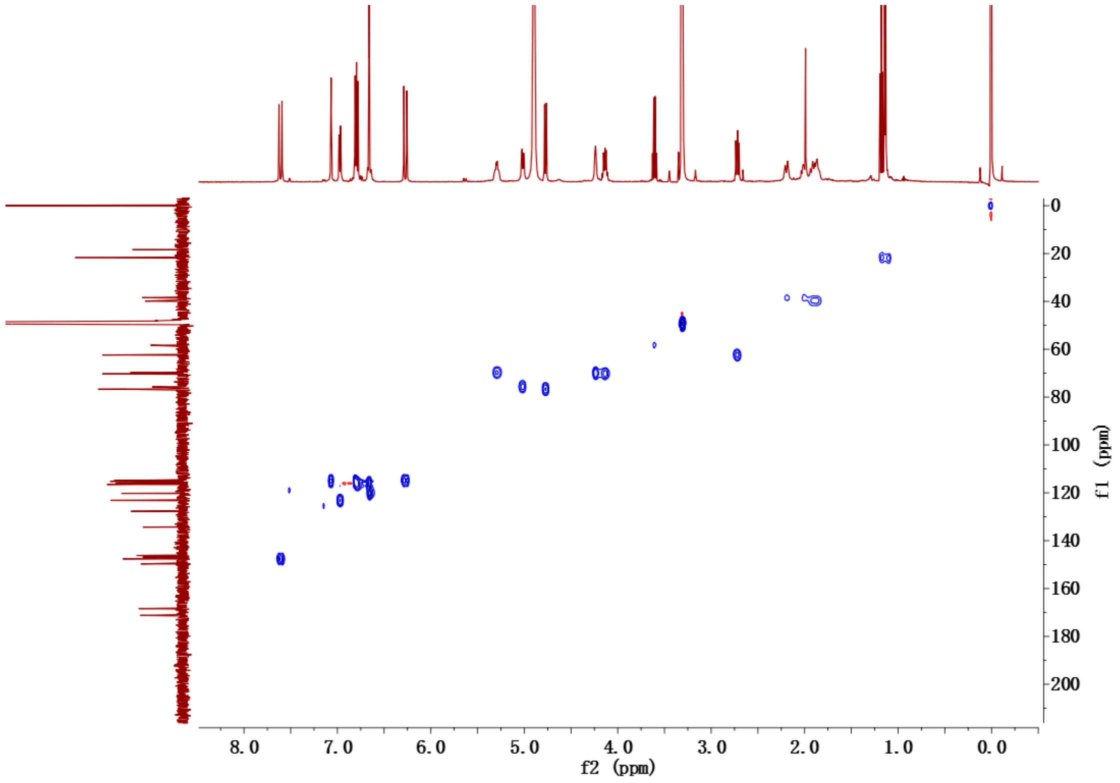

Figure S15. The HSQC Spectrum of Compound **2** in MeOH-d_4 (500 Hz)

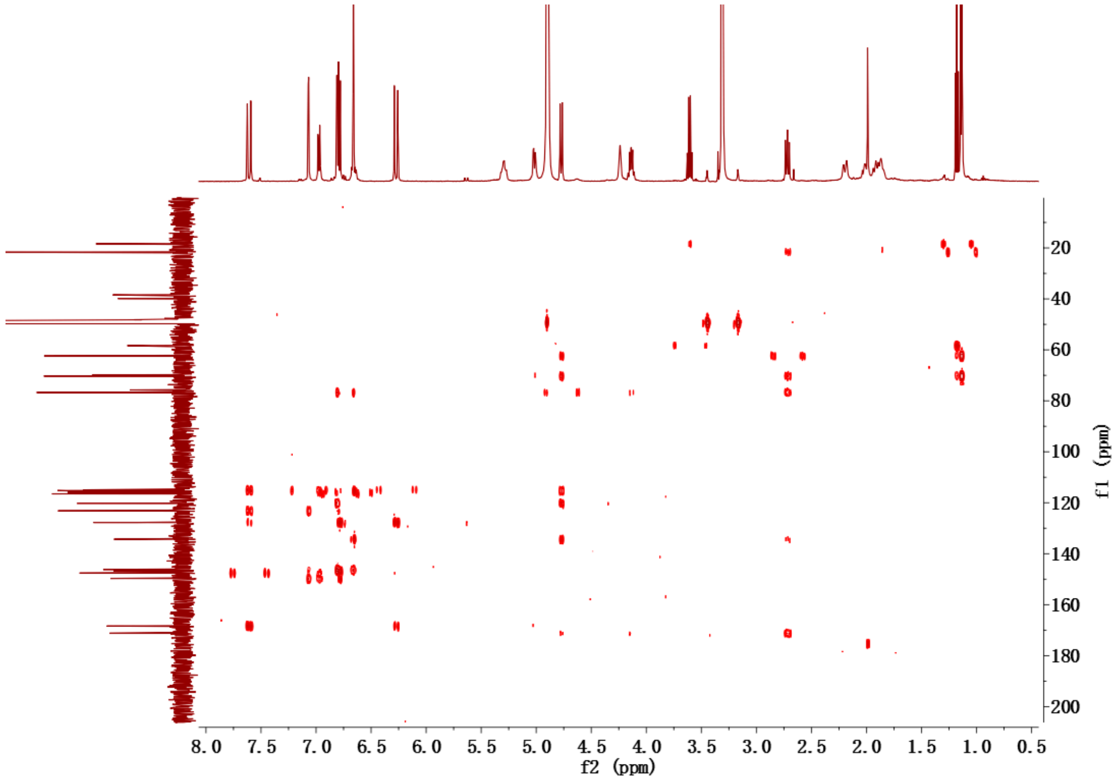

Figure S16. The HMBC Spectrum of Compound **2** in MeOH-d_4 (500 Hz)

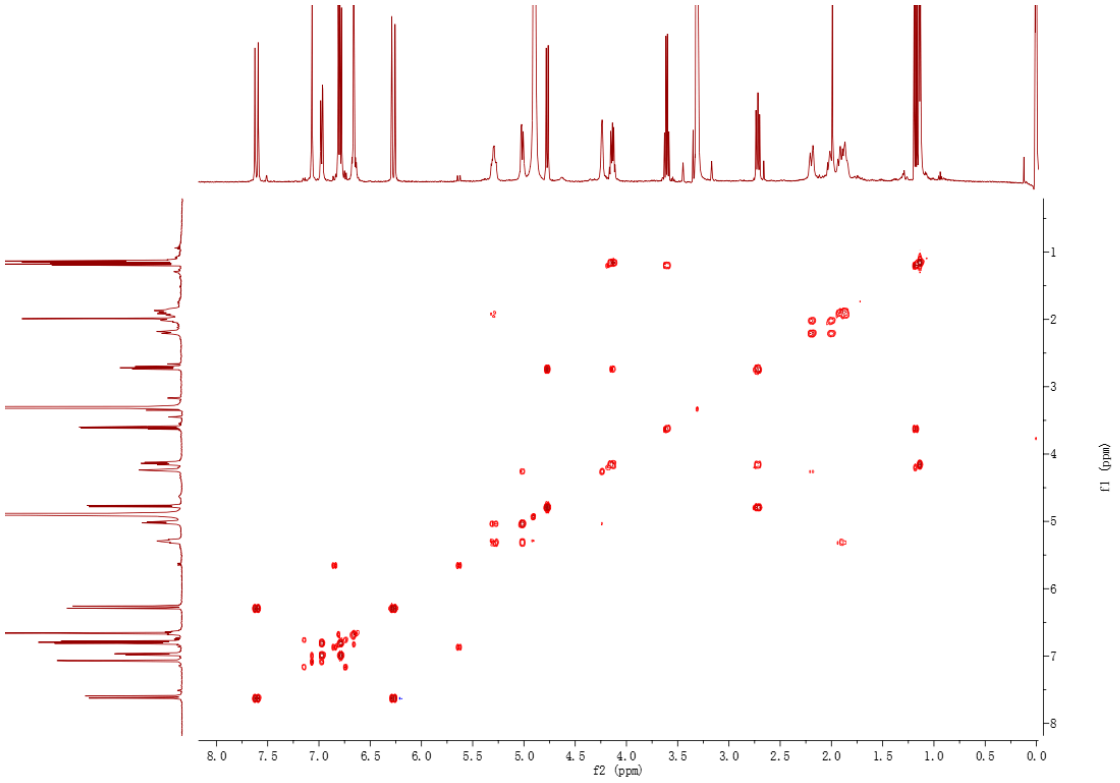

Figure S17. The HMBC Spectrum of Compound **2** in MeOH-*d*4 (500 Hz)

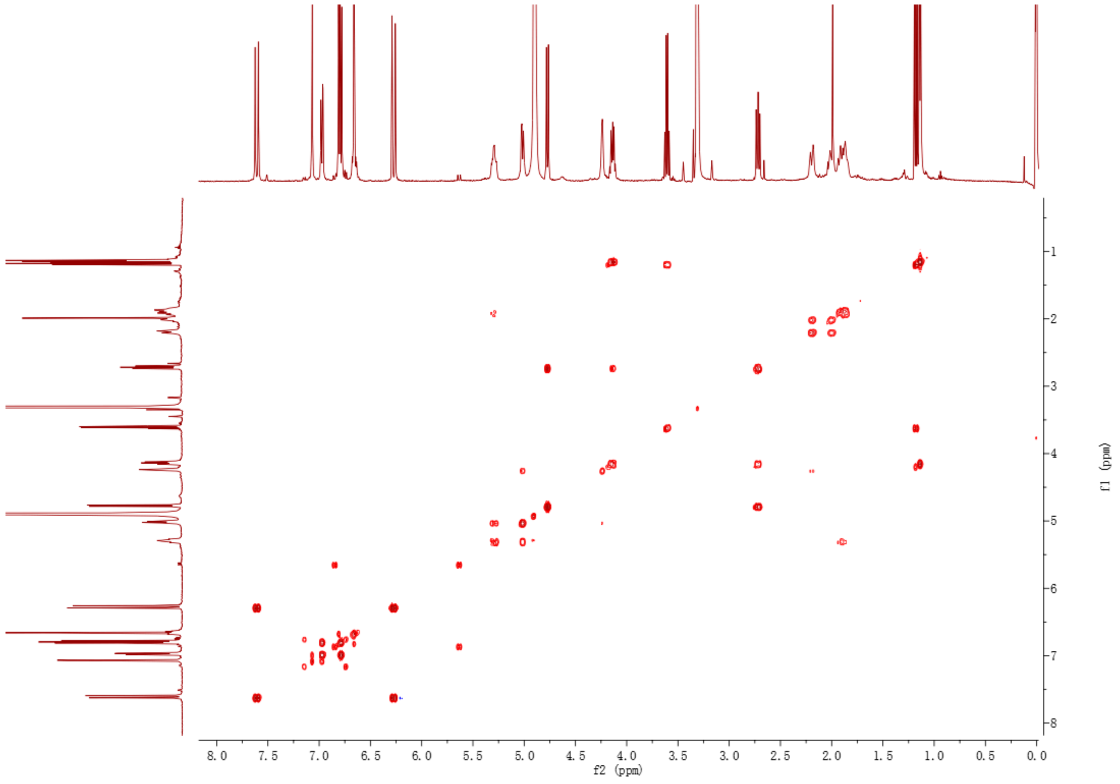

Figure S18. The ^{1}H-^{1}H COSY Spectrum of Compound **2** in MeOH-*d*4 (500 Hz)

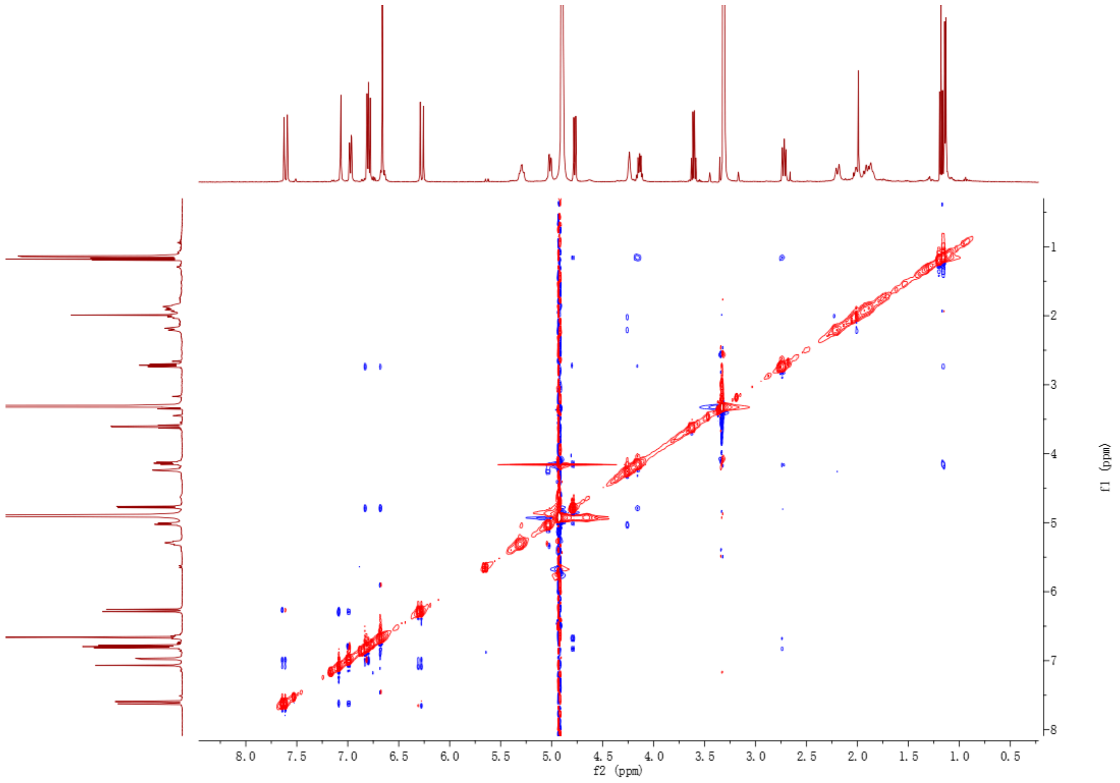

Figure S19. The ROESY Spectrum of Compound **2** in MeOH-*d*4 (500 Hz)

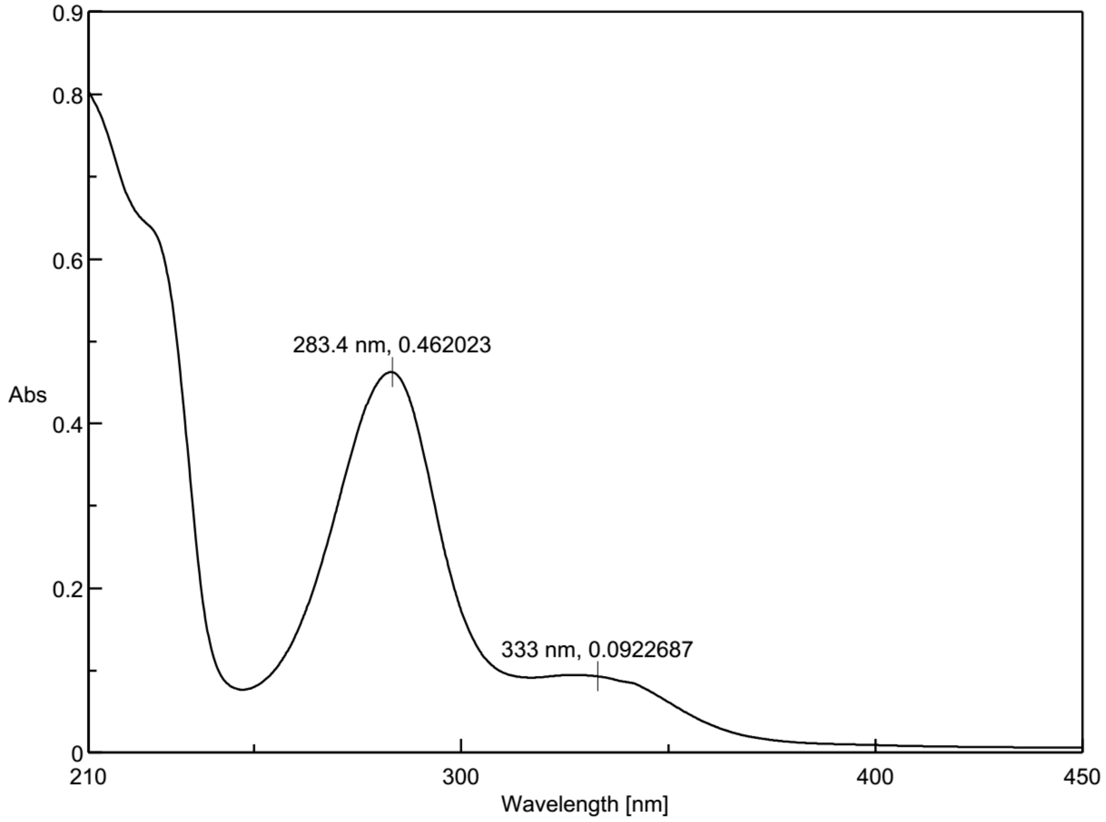

Figure S20. The UV Spectrum of compound **3** in MeOH

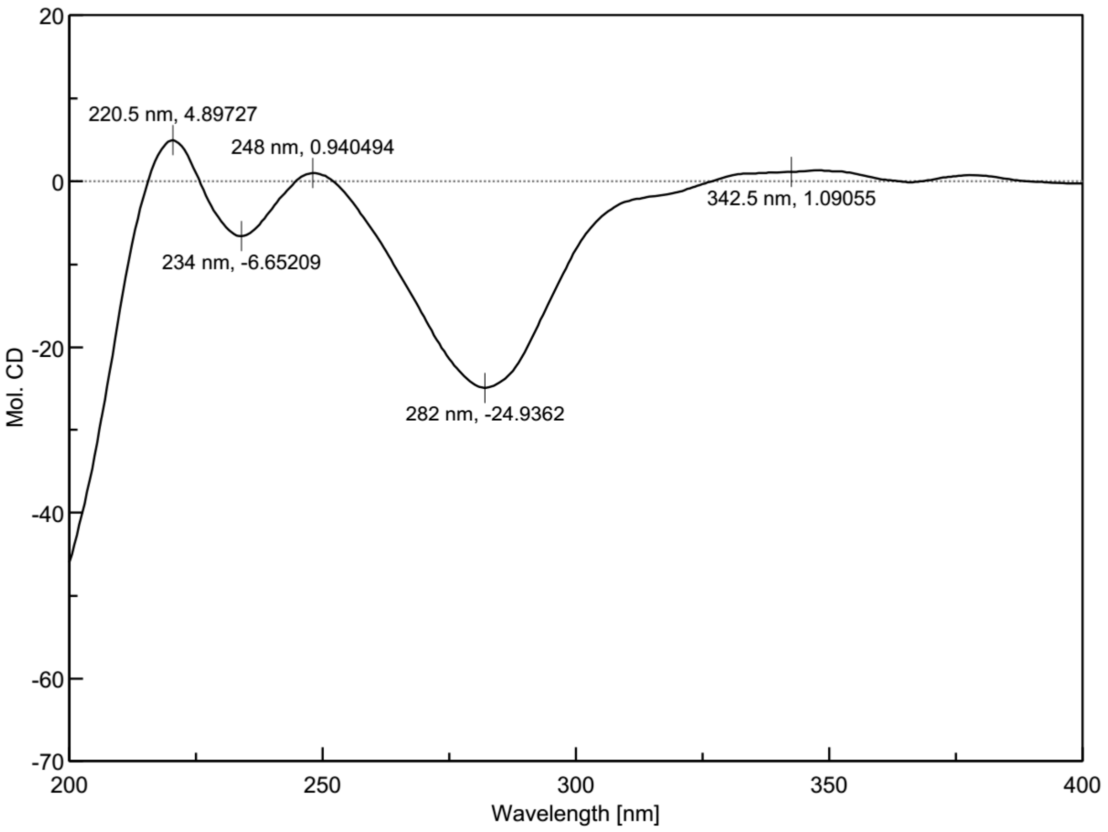

Figure S21. The ECD Spectrum of compound **3** in MeOH

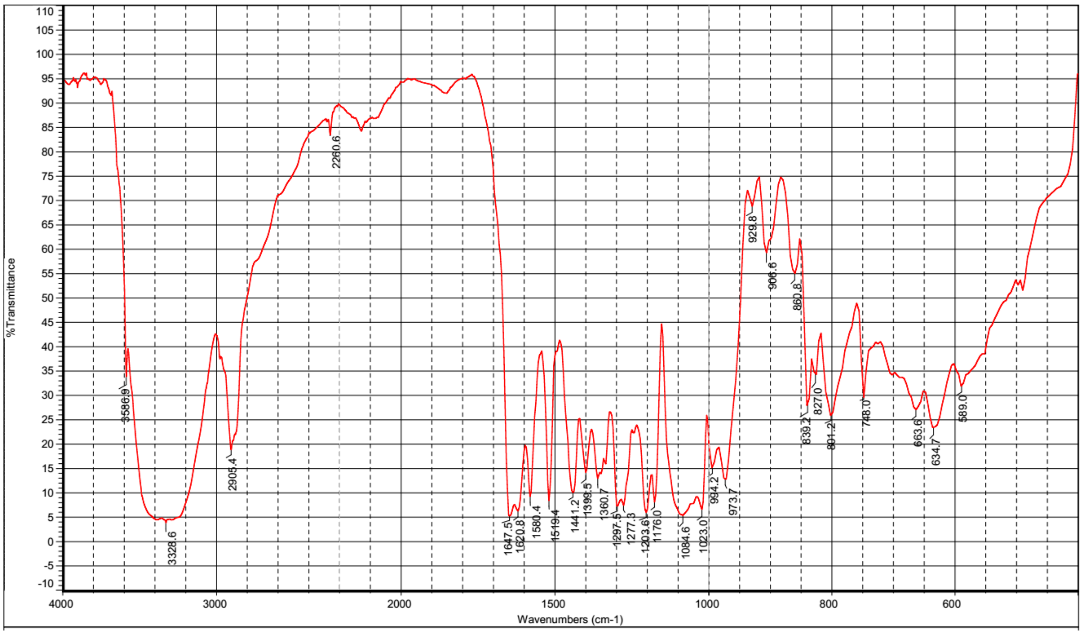

Figure S22. The IR Spectrum (KBr) of compound **3**

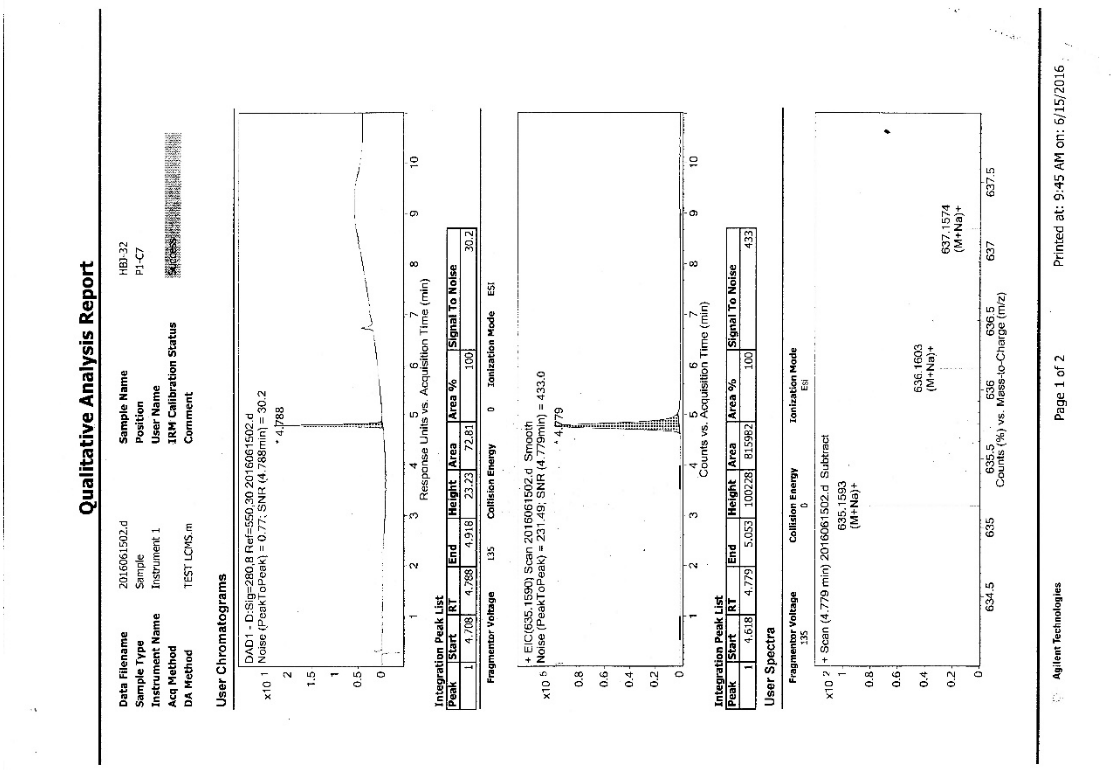

Figure S23. The HR-ESI-MS Data Spectrum (KBr) of compound **3**

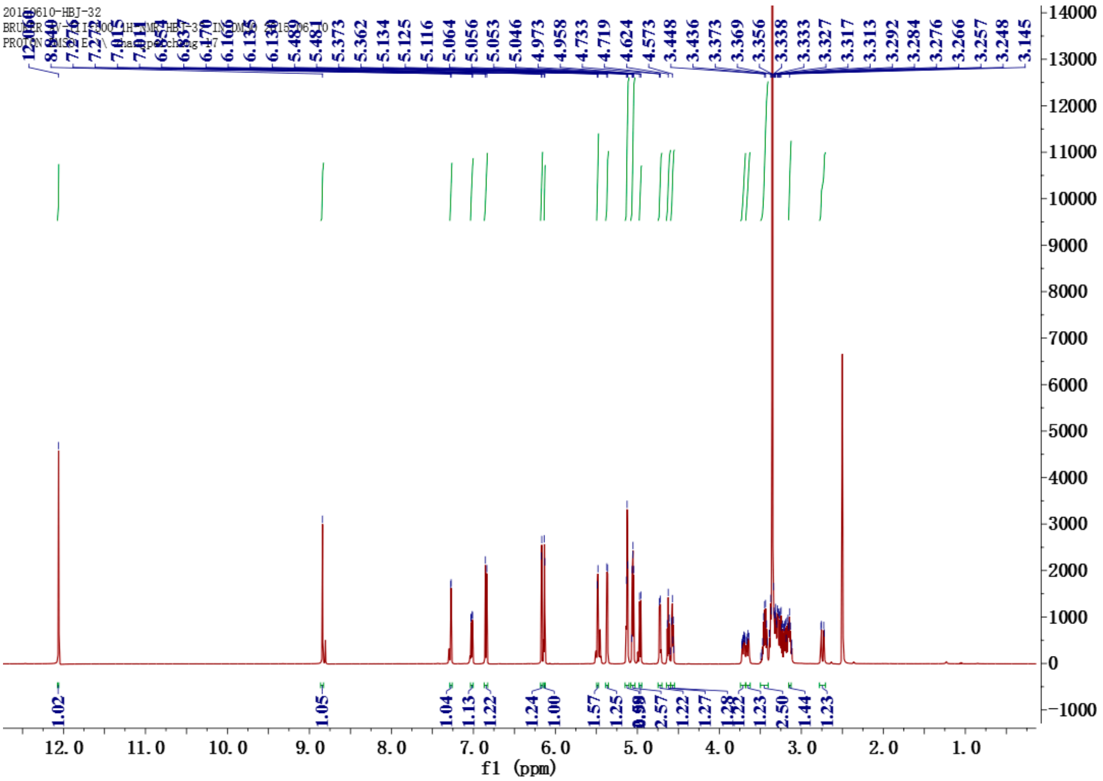

Figure S24. The ^{1}H NMR Spectrum of Compound **3** in DMSO-d_6 (500 Hz)

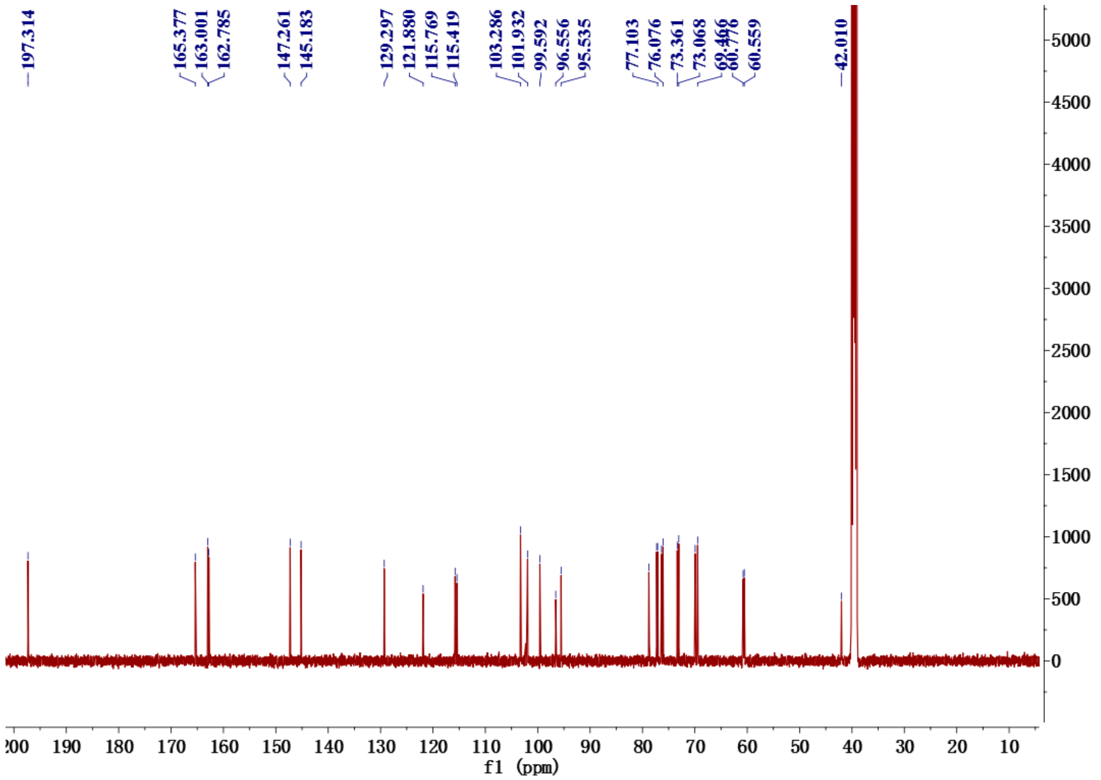

Figure S25. The ^{13}C NMR Spectrum of Compound **3** in DMSO-d_6 (125 Hz)

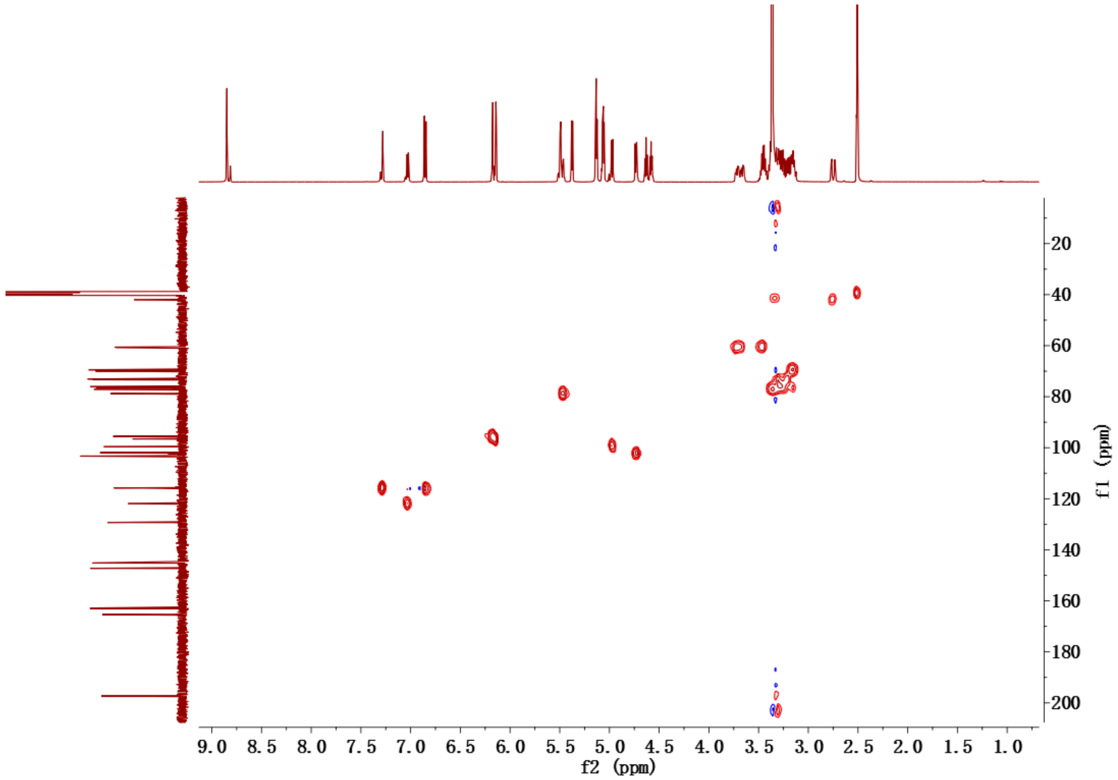

Figure S26. The HSQC Spectrum of Compound **3** in DMSO-d_6 (500 Hz)

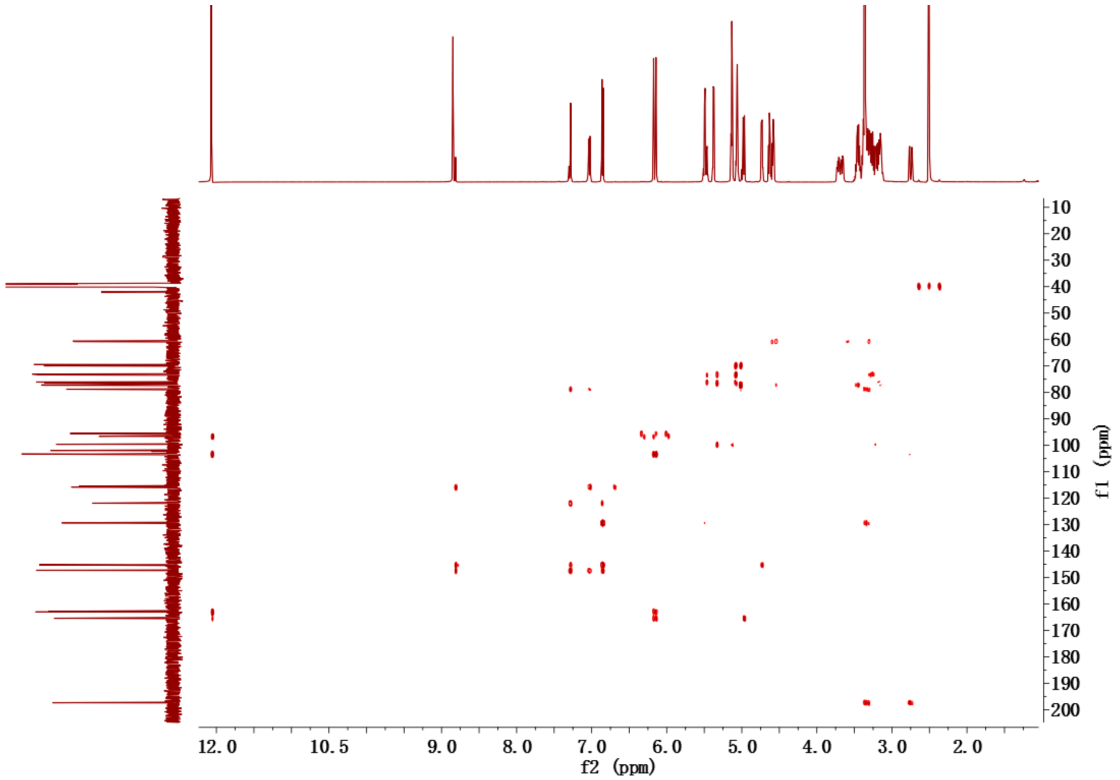

Figure S26. The HMBC Spectrum of Compound **3** in DMSO-d_6 (500 Hz)
